# An EEG-based framework for automated discrimination of conversion to Alzheimer’s disease in patients with amnestic mild cognitive impairment: an 18-month longitudinal study

**DOI:** 10.3389/fnagi.2024.1470836

**Published:** 2025-01-06

**Authors:** Yingfeng Ge, Jianan Yin, Caie Chen, Shuo Yang, Yuduan Han, Chonglong Ding, Jiaming Zheng, Yifan Zheng, Jinxin Zhang

**Affiliations:** ^1^Department of Medical Statistics, School of Public Health, Sun Yat-sen University, Guangzhou, China; ^2^Department of Clinical Medicine, Zhongshan School of Medicine, Sun Yat-sen University, Guangzhou, China; ^3^Department of Neurology, The First Affiliated Hospital, Sun Yat-sen University, Guangzhou, China

**Keywords:** amnestic mild cognitive impairment, EEG, discrimination, machine learning, longitudinal study

## Abstract

**Background:**

As a clinical precursor to Alzheimer’s disease (AD), amnestic mild cognitive impairment (aMCI) bears a considerably heightened risk of transitioning to AD compared to cognitively normal elders. Early prediction of whether aMCI will progress to AD is of paramount importance, as it can provide pivotal guidance for subsequent clinical interventions in an early and effective manner.

**Methods:**

A total of 107 aMCI cases were enrolled and their electroencephalogram (EEG) data were collected at the time of the initial diagnosis. During 18-month follow-up period, 42 individuals progressed to AD (PMCI), while 65 remained in the aMCI stage (SMCI). Spectral, nonlinear, and functional connectivity features were extracted from the EEG data, subjected to feature selection and dimensionality reduction, and then fed into various machine learning classifiers for discrimination. The performance of each model was assessed using 10-fold cross-validation and evaluated in terms of accuracy (ACC), area under the curve (AUC), sensitivity (SEN), specificity (SPE), positive predictive value (PPV), and F1-score.

**Results:**

Compared to SMCI patients, PMCI patients exhibit a trend of “high to low” frequency shift, decreased complexity, and a disconnection phenomenon in EEG signals. An epoch-based classification procedure, utilizing the extracted EEG features and *k*-nearest neighbor (KNN) classifier, achieved the ACC of 99.96%, AUC of 99.97%, SEN of 99.98%, SPE of 99.95%, PPV of 99.93%, and F1-score of 99.96%. Meanwhile, the subject-based classification procedure also demonstrated commendable performance, achieving an ACC of 78.37%, an AUC of 83.89%, SEN of 77.68%, SPE of 76.24%, PPV of 82.55%, and F1-score of 78.47%.

**Conclusion:**

Aiming to explore the EEG biomarkers with predictive value for AD in the early stages of aMCI, the proposed discriminant framework provided robust longitudinal evidence for the trajectory of the aMCI cases, aiding in the achievement of early diagnosis and proactive intervention.

## 1 Introduction

Dementia, resulting from various brain-related disorders and injuries, is a major cause of geriatric functional decline and caregiver reliance, ranking as the seventh leading cause of death globally (WHO, 2023). Currently, more than 55 million people are affected by dementia, with an annual increment of nearly 10 million new cases (Cao et al., 2020). AD is the most prevalent form of dementia and may account for 60–70% of cases (Alzheimer's Association, 2020). AD, distinguished by irreversible memory impairment, aphasia, apraxia, agnosia, and changes in personality and behavioral patterns, onsets insidiously with a prolonged course. Regrettably, effective pharmacological treatments for AD are not yet available. This underscores the critical importance of early screening and diagnosis so as to retard the progression and alleviate its disease burden.

Mild cognitive impairment (MCI) is a stage that falls between normal age-related cognitive decline and dementia, characterized by a subtle decline in cognitive functions that is not substantial enough to be classified as dementia. MCI may either stabilize or even improve over time, or progressively deteriorate into dementia (particularly AD), thus positioning it as a prodromal stage of AD. AMCI, characterized by memory dysfunction, is a subtype of MCI with an annual progression rate to AD ranging from 10 to 15% (Cai et al., 2020) and a lifetime conversion rate ranging from 75 to 80% (Gómez-Soria et al., 2021). Therefore, early and accurate prediction of the progression in aMCI stage becomes a crucial issue in managing the continuum of the disease and alleviating its burden.

The diagnosis of aMCI requires a combination of various examinations including biomarkers, neuroimaging, and neuropsychological assessments. This process is time-consuming, labor-intensive, and cost-prohibitive. Additionally, the insidious onset can be easily mistaken for age-related cognitive decline, thus significantly diminishing the detection rate of aMCI during clinical practice. As a non-invasive examination, EEG presents the benefits of convenience, cost-effectiveness, real-time diagnosis, and wide accessibility. It has been universally applied for the diagnosis and disease progression monitoring of aMCI. Compared to task-related EEG, resting-state EEG does not require examinees to perform complex instructions and actions, making it particularly suitable for elders with cognitive decline. Several studies have explored the spectral features of EEG in aMCI patients and identified indicators such as spectral power ratio (Flores-Sandoval et al., 2023), antero-posterior localization of alpha frequency (Huang et al., 2000), and spectral powers within the theta and delta bands (Roh et al., 2011) that exhibit favorable discriminatory capabilities, with an accuracy exceeding 80%. Simultaneously, researchers have investigated the EEG functional connectivity and graph theory features in aMCI patients, confirming the conjecture that aMCI serves as an intermediate stage between normal aging and AD (Frantzidis et al., 2014; Toth et al., 2014; Miraglia et al., 2016; Smailovic et al., 2022). Specifically, certain studies have conducted functional connectivity and graph theory analyses on the subdivisions of aMCI, namely stable MCI (SMCI) and MCI progress to AD (PMCI), revealing differences between the two groups and achieving promising predictive outcomes (Vecchio et al., 2018; Miraglia et al., 2020). Serving as an intermediate stage between normal aging and AD, aMCI exhibits considerable EEG variability, reflecting the heterogeneity within the aMCI population.

Recently, there has been widespread use of machine learning methods for discriminant diagnosis through EEG data in patients with AD and MCI. However, few studies have specifically targeted the aMCI population. Li et al. (2021) combined the characteristics of brain functional network with support vector machine classifier in aMCI and healthy controls (HC), achieved an accuracy of 86.60%. The same research team (Li et al., 2022) incorporated spectral entropy features into a convolutional neural network (CNN) model, attaining an accuracy of 94.64% in aMCI and HC. Kim et al. (2022) explored different patterns of functional networks between aMCI and non-aMCI using EEG graph theoretical analysis, the naive Bayes algorithm classified aMCI and non-aMCI with 89% accuracy. Farina et al. (2020) employed penalized logistic regression models to identified the power and functional connectivity features of EEG in AD, aMCI, and HC populations, but the accuracy remained unstable across various combinations of features. The aforementioned studies all treated aMCI as a unified discrimination category, without conducting follow-up assessments of the disease progression within aMCI, which would allow for further subdivision into SMCI and PMCI and subsequently exploration of EEG differences between these two subgroups with imperative longitudinal study (Mammone et al., 2018; Ruiz-Gomez et al., 2018; Ding et al., 2022; Jiang et al., 2022; Kim et al., 2022; Lassi et al., 2023; Wijaya et al., 2023). However, early prediction of whether aMCI will progress to AD is of paramount importance, as it aids in guiding subsequent interventions involving medications, lifestyle, rehabilitation, and healthcare in an advanced and effective manner. Currently, there is a scarcity of longitudinal studies concerning aMCI cases, as well as a lack of research applying machine learning methods with constrained EEG features to disease discrimination and prediction in SMCI and PMCI subgroups.

This study recruited aMCI patients and collected the EEG data at the time of initial diagnosis. After an 18-month follow-up period, patients were categorized into SMCI and PMCI groups based on whether they progressed to AD, which was in alignment with definitions from prior research (Vecchio et al., 2018). By comprehensively extracting EEG spectral, nonlinear, and functional connectivity features, we conducted feature selection and dimensionality reduction on extracted features. Subsequently, selected features were integrated into different machine learning classifiers for discrimination, to explore EEG biomarkers with potential for early prediction. Utilizing the afore mentioned framework, we systematically extracted EEG features with excellent discriminant ability between the SMCI and PMCI populations and to discern the heterogeneity in disease progression among individuals with aMCI, enabling the early identification of progressing cases and facilitating the implementation of three levels of prevention, which conducting prospective exploration for follow-up study in the future.

## 2 Materials and methods

**Figure 1:**
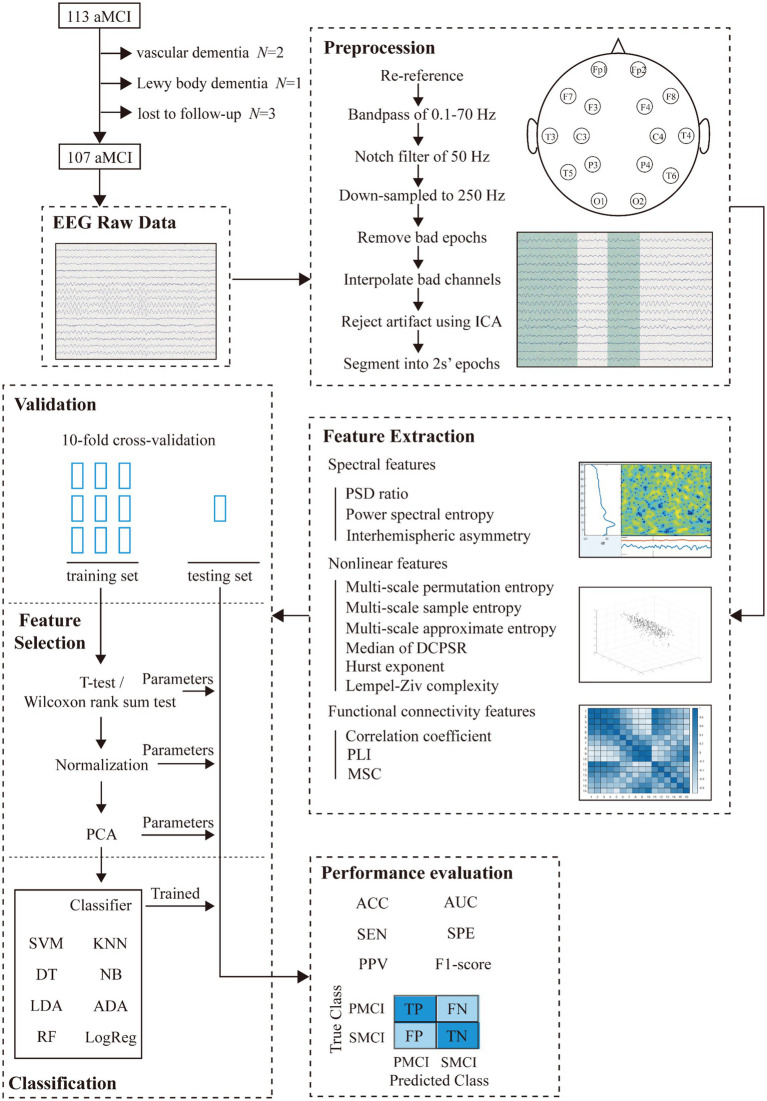
Study design.

The discriminant framework of this study design was shown in Figure 1, which consisted of five main steps: EEG data acquisition, EEG preprocessing, feature extraction, classification, and evaluation.

### 2.1 Participants

Between September 1, 2021 and April 30, 2022, we recruited a total of 113 aMCI patients from the Memory Clinic Unit of the First Affiliated Hospital of Sun Yat-sen University (SYSU), and 107 patients completed the follow-up without any censored data. We collected their raw EEG data at the time of initial diagnosis and conducted an 18-month follow-up for each patient to obtain clinical outcomes after 18 months. The diagnosis of aMCI was based on the Petersen 2011 criteria (Petersen, 2004), and made in a blinded manner with respect to the EEG examination. The inclusion criteria for this study were as follows: (1) age of 50 years and above, (2) memory complaint usually corroborated by an informant, (3) objective memory impairment for age, (4) essentially preserved general cognitive function, (5) largely intact functional activities. The exclusion criteria were: other forms of dementia or accompanying Parkinson’s disease, epilepsy, psychiatric disorders, and serious organic disease. Among 107 aMCI cases, 42 individuals were diagnosed with AD after 18 months, while 65 individuals remained in the aMCI stage. Next, the 107 aMCI patients were divided into two groups: PMCI and SMCI. The diagnosis of AD was based on the criteria provided by the National Institute on Aging and the Alzheimer’s Association (NIA-AA) in 2011 (McKhann et al., 2011). All disease diagnoses in this study were accomplished by experienced neurologists. This study adhered to the Helsinki Declaration and was approved by the Ethics Committee of the School of Public Health, Sun Yat-sen University (2021-No.081). The demographic information of the patients was shown in Table 1.

**Table 1:** The demographic characteristics of participants.

Variable	SMCI (*n* = 65)	PMCI (*n* = 42)	Statistics
Age (years)	68.85 ± 8.76	68.17 ± 8.08	*t* = 0.40, *p* = 0.69
Gender (male/female)	24/41	22/20	$\chi^{2}$ =2.49, *p* = 0.12
MMSE (scores)	23.58 ± 2.87	23.55 ± 2.47	*t* = 0.07, *p* = 0.95
MoCA (scores)	19.31 ± 3.36	18.52 ± 3.01	*t* = 1.23, *p* = 0.22
Type of aMCI (single/multiple)	27/38	17/25	$\chi^{2}$ =0.01, *p* = 0.91
Diabetes (yes/no)	25/40	13/29	$\chi^{2}$ =0.63, *p* = 0.43
Hypertension (yes/no)	41/24	23/19	$\chi^{2}$ =0.73, *p* = 0.39
Level of education			$\chi^{2}$ =0.03, *p* = 0.99
Primary education	10	6	
Secondary education	49	32	
Higher education	6	4	

MMSE, Mini-Mental State Examination; MoCA, Montreal Cognitive Assessment.

### 2.2 EEG data acquisition

Resting-state EEG was recorded using the Nicolet EEG machine system (Natus Medical Inc., San Carlos, CA) with a sampling rate of 500 Hz. Electrodes were placed according to the 10–20 international system, with a total of 16 channels (Fp1, Fp2, F3, F4, C3, C4, P3, P4, O1, O2, F7, F8, T3, T4, T5, and T6). All patients were right-handed, and sufficient sleep was ensured the night before the EEG collection. During the recording, patients were instructed to maintain a comfortable seated posture with their eyes closed for 5 min. EEG technicians continuously monitored the EEG traces and promptly alerted participants if any signs of drowsiness were detected.

### 2.3 EEG preprocessing

EEG signals are susceptible to various artifacts, highlighting the importance of preprocessing prior to analysis. Firstly, the raw EEG data were re-referenced using an average reference, and a finite impulse response (FIR) band-pass filter was applied to filter the EEG signals within the range of 0.1–70 Hz. Also, a notch filter was used to eliminate the 50 Hz power line interference. The EEG signals were subsequently down-sampled to 250 Hz. After joint screening by two experienced EEG examiners, bad epochs were removed and bad channels were interpolated. Then, 20-s segments of continuous EEG signals with clear background rhythms and minimal interference were selected. Following, we conducted independent component analysis (ICA) to remove common artifacts such as blinks, eye movements, and cardiac interference. Finally, the EEG signals were segmented into non-overlapping 2-s epochs for subsequent feature extraction. The above preprocessing steps were all performed using the EEGLAB toolbox (Delorme and Makeig, 2004) in MATLAB (R2023a, MathWorks).

### 2.4 Feature extraction

For each 2 s EEG epoch, we extracted features in three feature sets: spectral, nonlinear, and functional connectivity.

#### 2.4.1 Spectral feature

Using Welch’s power spectral density (PSD) estimation (Alam et al., 2020), we transformed the preprocessed EEG signals from the time domain into the frequency domain and divided them into the following five subbands: delta (0.5–4 Hz), theta (4–8 Hz), alpha (8–13 Hz), beta (13–30 Hz), and gamma (30–45 Hz).

(1) Power spectral density ratio (PSD ratio): Considering the variation in absolute PSD values among different patients, we calculated the relative PSD values within the aforementioned subbands for each patient (see Equations 1–5) resulting in the following five ratios:

(1) Ratio1=delta/alpha

(2) Ratio2=theta/alpha

(3) $$\mathrm{Ratio}3=\mathrm{delta}/\left(\mathrm{alpha}+\mathrm{beta}\right)$$

(4) $$\mathrm{Ratio}4=\mathrm{theta}/\left(\mathrm{alpha}+\mathrm{beta}\right)$$

(5) $$\mathrm{Ratio}5=\left(\mathrm{delta}+\mathrm{theta}\right)/\left(\mathrm{alpha}+\mathrm{beta}+\mathrm{gamma}\right)$$

(2) Power spectral density entropy (PSDE): In each subband, a sequence of PSD values can be obtained. We used the Shannon entropy method to assess the level of disorder in this sequence of PSD values (Li et al., 2023). Assuming there are PSD series with *N* values within the subband, the PSDE was calculated as follows:

(6) $$E=-\overset{N}{\underset{i=1}{\sum }}p_{i}\log p_{i}$$

where *E* and $p_{i}$ represent the PSDE of the signal and the probability of having the i sample in the signal, respectively (see Equation 6).

(3) Interhemispheric asymmetry (IA): IA quantifies the disparity in PSD between the left and right channels, reflecting differences in the distribution of PSD values in symmetrical brain regions. IA is calculated as follows:

(7) $$IA=\log\left(PSD_{lc}\right)-\log\left(PSD_{rc}\right)$$

where IA, $PSD_{lc}$ , and $PSD_{rc}$ stand for the interhemispheric asymmetry, the PSD in the left hemisphere, and the PSD in the right hemisphere, respectively. We computed the IA values for a total of eight pairs (Fp1-Fp2, F3-F4, C3-C4, P3-P4, O1-O2, F7-F8, T3-T4, T5-T6) of channels across five subbands (see Equation 7).

#### 2.4.2 Nonlinear feature

We extracted the following six nonlinear features to capture the nonlinear characteristics of the EEG signals in aMCI patients. The specific formulas can be found in Appendix A.

(1) Multi-scale permutation entropy (PE): PE is an efficient quantitative complexity measure that explores the local order structure of a dynamic time series (Bandt and Pompe, 2002), particularly in EEG signals from MCI and AD patients (Siuly et al., 2020; Şeker et al., 2021). Multi-scale PE provides a multiscale perspective on signal complexity, facilitating the investigation of these neurological conditions (PARK et al., 2007; Wu et al., 2013; Deng et al., 2017). Our study calculated the PE for scales ranging from 1 to 10 (Busa and van Emmerik, 2016).
(2) Multi-scale approximate entropy (AE): AE is a metric that quantifies the repetitiveness of a time series, capturing its irregular and chaotic nature by assessing the recurrence of patterns within the time series, including the EEG signals in MCI and AD cases (Abásolo et al., 2008; Nimmy John et al., 2019). In our study, AE was calculated for scales ranging from 1 to 10.
(3) Multi-scale sample entropy (SE): The SE is proposed by Richman and Moorman (2000) as an improvement over AE, addressing the bias present in AE. Recently, SE has been extensively utilized for feature extraction in patients with MCI and AD (Tsai et al., 2012; Ruiz-Gomez et al., 2018). Also, our study calculated the SE for scales ranging from 1 to 10.
(4) Lempel-Ziv complexity (LZ): LZ, a nonlinear feature in EEG signal analysis, exhibits distinctive characteristics in MCI and AD patients, highlighting encoding intricacies that could reveal disease-related patterns (Abásolo et al., 2006; Liu et al., 2016; Ruiz-Gomez et al., 2018). We selected the average of the EEG signal as the coarse-graining method for LZ analysis in this study.
(5) Hurst exponent: The Hurst exponent quantifies the long-term memory or self-similarity of a time series, indicating whether it tends to exhibit persistent trends, mean reversion, or random behavior. This is valuable for distinguishing different EEG activity patterns and monitoring the temporal characteristics of EEG signals in MCI and AD patients (Nimmy John et al., 2018; Amezquita-Sanchez et al., 2019).

The aforementioned five nonlinear metrics all reflect the complexity of the EEG signals, with higher values indicating greater variability in the EEG signal, and vice versa.

(6) Median distance from the centroid of phase space reconstruction (M-DCPSR): Phase space reconstruction (PSR) is applied in EEG research to unveil the nonlinear dynamical properties and spatiotemporal relationships of brain electrical activity (Lee et al., 2014; Kaur et al., 2020). We innovatively propose M-DCPSR to unveil the nonlinear characteristics of EEG in the aMCI population. Firstly, we set the embedding dimension of PSR as *m* = 3 and determined the lag of the time series ( τ ) using the autocorrelation function. Subsequently, the three-dimensional coordinates of the time series in the phase space were constructed based on τ . Next, the centroid of the structure formed by all points in the phase space was computed, and the Euclidean distance between each point and the centroid was calculated. Finally, we computed the median of these Euclidean distances, resulting in the M-DCPSR for the given time series.

#### 2.4.3 Functional connectivity feature

We extracted three functional connectivity metrics to measure the consistency of EEG signals across channels in aMCI patients. The specific formulas can be found in Appendix A.

(1) Correlation coefficient: The Pearson correlation coefficient (*r*) can measure linear relationships in EEG connectivity research. The equation for calculating *r* between two signals *X* and *Y* is:

(8) $$r=\frac{\sum_{i=1}^{n}\left(X_{i}-\bar{X}\right)\left(Y_{i}-\bar{Y}\right)}{\sqrt{\sum_{i=1}^{n}{\left(X_{i}-\bar{X}\right)}^{2}\sum_{i=1}^{n}{\left(Y_{i}-\bar{Y}\right)}^{2}}}$$

where *n* is the number of data points, $\bar{X}$ and $\bar{Y}$ are the means of signals *X* and *Y*, respectively (see Equation 8).

(2) Phase lag index (PLI): PLI, which is used to measure the degree of phase synchronization between two signals, can exclude the influences of volume conduction in EEG signals. It is commonly employed as a functional connectivity feature in MCI and AD patients (Núñez et al., 2019; Nobukawa et al., 2020; Kuang et al., 2022). PLI values range from 0 to 1. A PLI of zero indicates either no coupling or coupling with a phase difference centered around 0 or *π*. A PLI of 1 indicates perfect phase locking at a value different from 0 or π.
(3) Magnitude squared coherence (MSC): MSC is frequently employed in EEG connectivity studies to assess the dependence between two signals. The MSC value ranges from 0 to 1. An MSC of 0 indicates no linear dependence between the two signals. A larger MSC value suggests a higher degree of statistical dependence between the two signals.

The total number of extracted features can be found in Appendix B.

### 2.5 Classification and validation

We employed eight commonly used machine learning classifiers for binary discrimination in AD Spectrum (Perez-Valero et al., 2021; Tzimourta et al., 2021; Rossini et al., 2022), including support vector machine (SVM), decision tree (DT), naive Bayes (NB), linear discriminant analysis (LDA), AdaBoost (ADA), *k*-nearest neighbor (KNN), random forest (RF), and logistic regression (LogReg). The detailed descriptions of eight classifiers can be found in Appendix C. All the parameters for machine learning models were set to the default settings in MATLAB. All 2 s EEG epochs were divided into training and testing sets using a 10-fold cross-validation approach at the subject level, ensuring that EEG epochs from the same participant were not simultaneously included in both the training and testing sets. We conducted feature selection and dimensionality reduction on the aforementioned extracted features. Firstly, we employed two-sample *t*-test and Wilcoxon rank-sum test to select features with statistical significance between the two groups in the training set. Then, the selected features were standardized and subjected to principal component analysis (PCA) for dimensionality reduction, extracting principal components that contribute to 95.00% cumulative variance. Next, we applied the feature selection parameters from the training set to the testing set, to prevent data leakage issues in machine learning.

Finally, we accessed the classification performance of the machine learning model using six metrics: sensitivity (SEN), specificity (SPE), positive predictive value (PPV), F1-score, accuracy (ACC), and area under the curve (AUC) for the receiver operating characteristic curve (see Equations 9–14). The formula for the previously mentioned metrics is as follows:


(9) $$ACC=\frac{TP+TN}{TP+FN+TN+FP}$$

(10) $$SEN=\frac{TP}{TP+FN}$$

(11) $$SPE=\frac{TN}{FP+TN}$$

(12) $$PPV=\frac{TP}{TP+FP}$$

(13) $$F1-\mathrm{score}=\frac{2TP}{2TP+FP+FN}$$

(14) $$AUC=\frac{\sum_{\mathrm{in}s_{i}\in \mathrm{positiveclass}}\mathrm{ran}k_{\mathrm{in}s_{i}}-\frac{M\times \left(M+1\right)}{2}}{M\times N}$$

where *M*, *N* are the number of positive sample and negative sample, separately (Hanley and McNeil, 1982; Cortes and Mohri, 2003). *TP* is the number of PMCI cases that are correctly predicted, *FN* is the number of PMCI cases that are incorrectly predicted as SMCI samples, *FP* is the number of SMCI cases that are incorrectly predicted as PMC cases, and *TN* is the number of SMCI samples that are correctly predicted.

## 3 Results

In this section, we firstly presented the statistical differences of three feature sets in SMCI and PMCI cases. Since the assumptions of parametric tests were not met for these feature sets, we employed two-sample Wilcoxon rank-sum tests to explore the statistical differences of aforementioned features between the two groups. Finally, we presented the discriminant performance of different classifiers.

### 3.1 Spectral features

**Figure 2:**
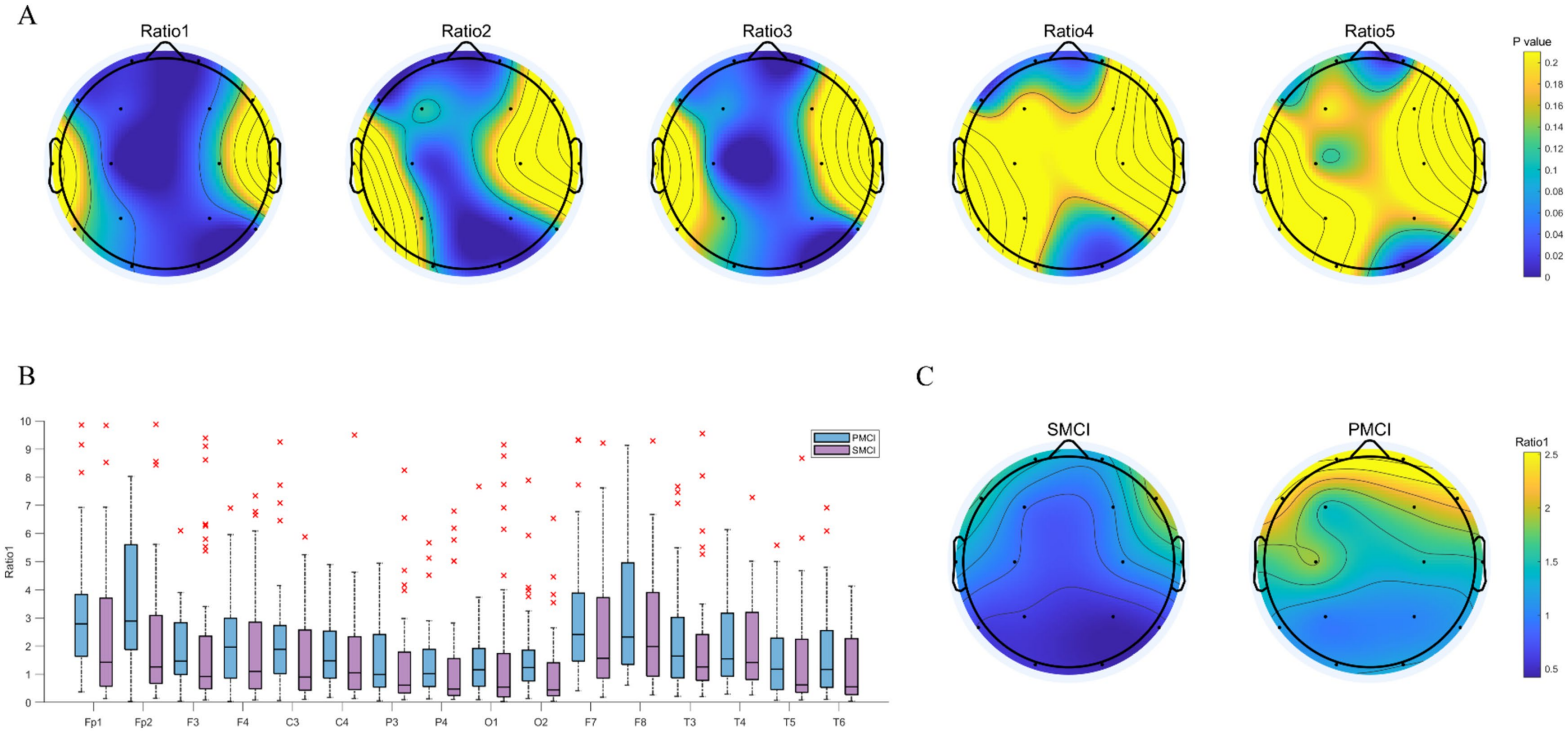
The combination chart of statistical differences for PSD ratios in SMCI and PMCI groups. **(A)** The EEG topoplot in terms of *p*-value of five PSD ratios between the SMCI and PMCI groups using the Wilcoxon Rank-Sum Test. **(B)** The boxplot of PSD ratio1 for the SMCI and PMCI groups. The horizontal axis represents 16 channels, and the vertical axis represents the values of PSD ratio1. **(C)** The EEG topoplot in terms of the mean of PSD ratio1 within the SMCI and PMCI groups.

As shown in Figure 2, the disparities in PSD ratio1, PSD ratio2, and PSD ratio3 between SMCI and PMCI cases were more significant compared to PSD ratio4 and PSD ratio5. The PSD ratio1 exhibited the most pronounced distinguished capability between the two groups, followed by PSD ratio3 and PSD ratio2. In the frontal, central, parietal, and occipital regions, the value of PSD ratio1 in the PMCI group was noticeably higher than that in the SMCI group.

**Figure 3:**
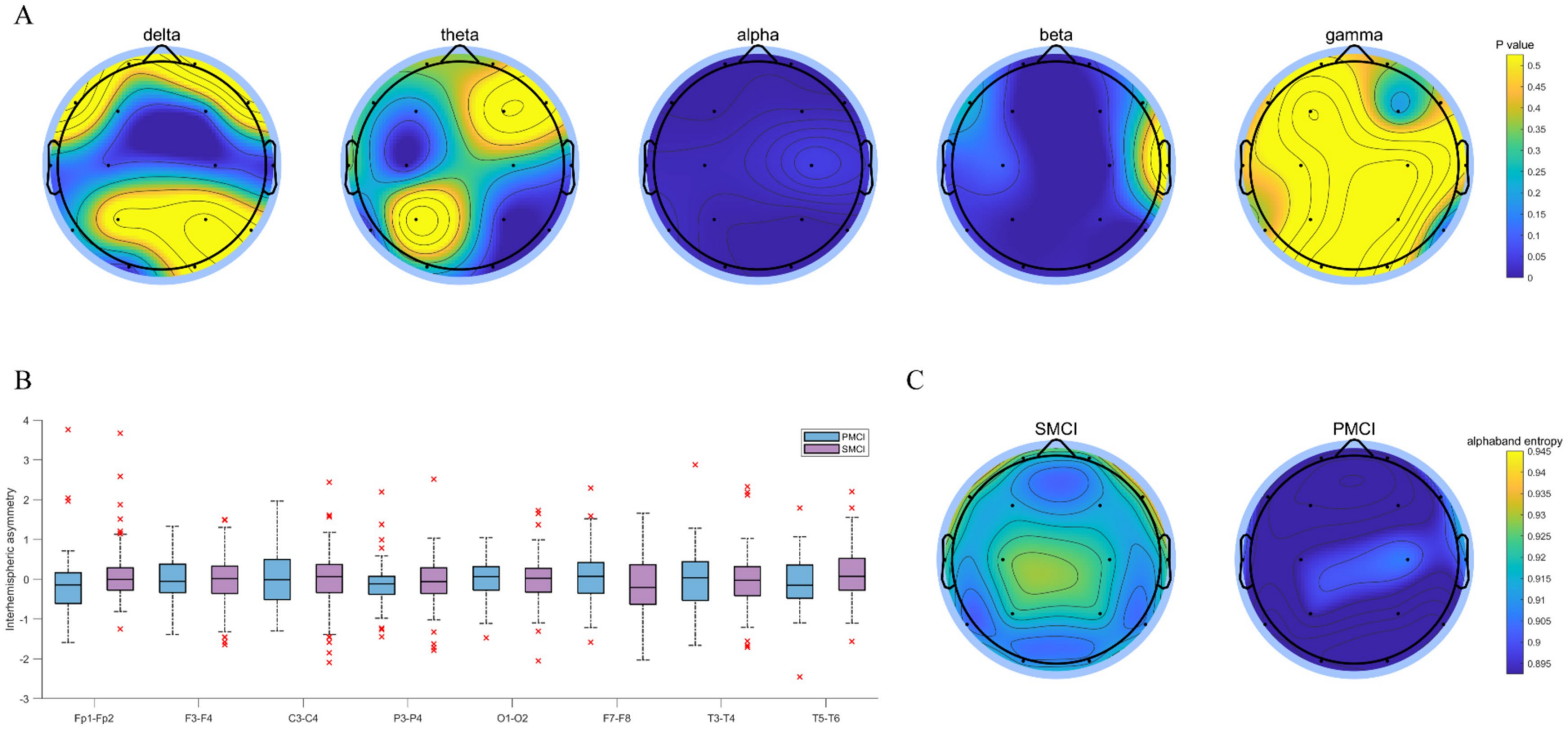
The combination chart of statistical differences for PSDE and IA in SMCI and PMCI groups. **(A)** The EEG topoplot in terms of *p*-value of five PSDE between the SMCI and PMCI groups using the Wilcoxon Rank-Sum Test. **(B)** The boxplot of IA in the delta band for the SMCI and PMCI groups. The horizontal axis represents 8 channel pairs, and the vertical axis represents the values of IA. **(C)** The EEG topoplot in terms of the mean of PSDE in the alpha band within the SMCI and PMCI groups.

As illustrated in Figure 3, the differences in PSDE in the alpha and beta bands were more significant than those in the delta, theta, and gamma bands between SMCI and PMCI cases. The PSDE in the alpha band exhibited the best distinguished capability between the two groups, followed by the beta band. The PSDE in the alpha band were notably lower in all brain regions in the PMCI cases compared to the SMCI cases. However, there was no significant difference between SMCI and PMCI in IA in the delta band. The same results were observed in IA with the four other frequency bands as well (see Appendix B).

### 3.2 Nonlinear feature

**Figure 4:**
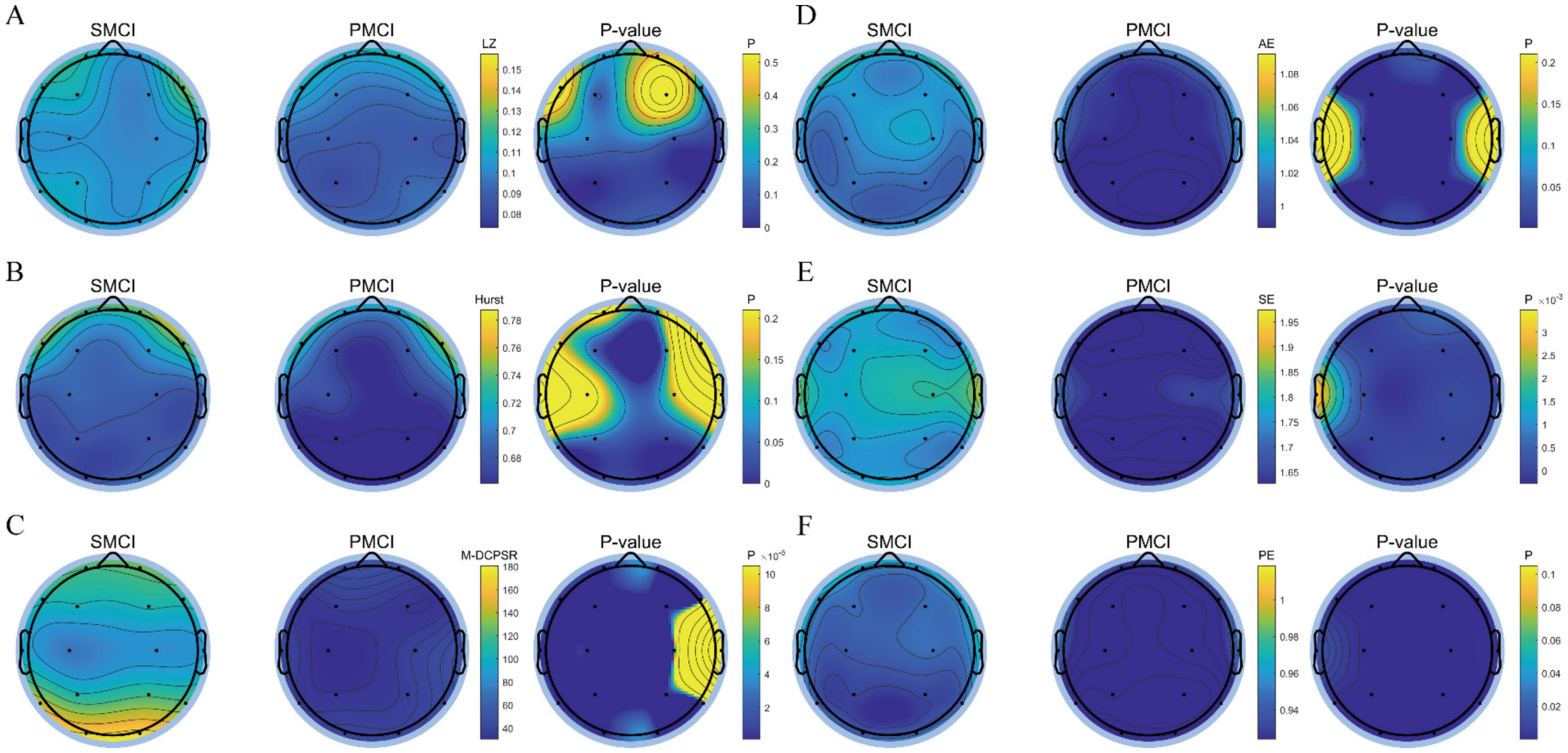
The topoplot of statistical differences for nonlinear features in SMCI and PMCI groups. **(A)** LZ; **(B)** Hurst exponent; **(C)** M-DCPSR; **(D)** AE, scale = 2; **(E)** SE, scale = 2; **(F)** PE, scale = 2. The first two columns of each subplot represent the EEG topoplot in terms of the mean of various nonlinear features within the SMCI and PMCI groups, respectively. The last column shows the EEG topoplot in terms of *p*-value of nonlinear features between the SMCI and PMCI groups using the Wilcoxon Rank-Sum Test.

As shown in Figure 4, the differences in SE, PE, and M-DCPSR between SMCI and PMCI cases were more significant compared to AE, LZ, and the Hurst exponent. Compared to the SMCI group, the PMCI group exhibits lower values of SE, PE, and M-DCPSR in all brain regions. AE exhibited better discriminant performance in the frontal, parietal, and occipital regions; the Hurst exponent demonstrated better discriminant performance in the frontal and occipital regions. However, LZ showed limited distinguished efficacy between SMCI and PMCI patients.

### 3.3 Functional connectivity feature

**Figure 5:**
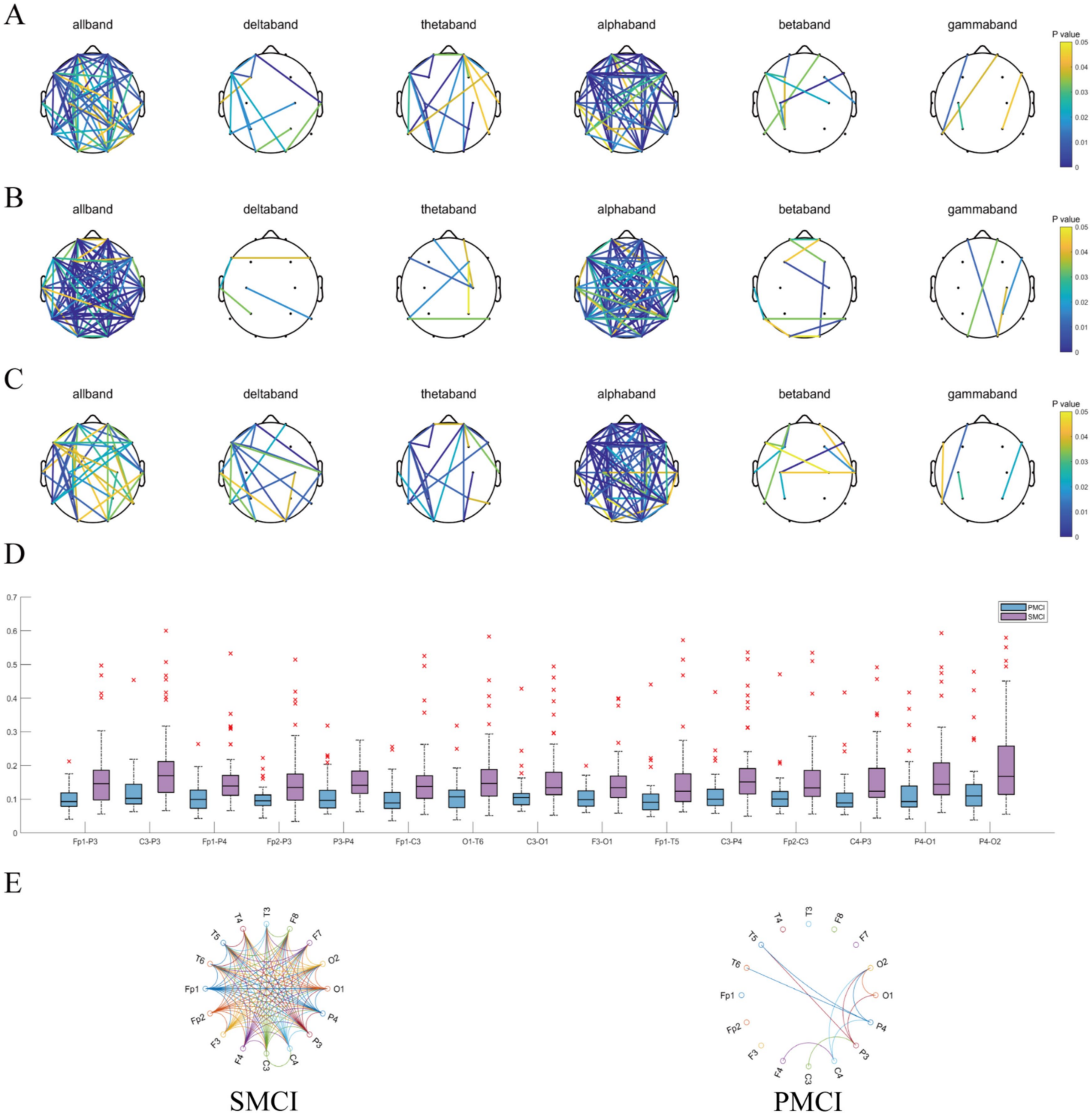
The combination chart of statistical differences for functional connectivity features in SMCI and PMCI groups. **(A)** Pearson correlation coefficient; **(B)** PLI; **(C)** MSC. In each subplot, the connections have statistically significance between the SMCI and PMCI groups, with color variations indicating the magnitude of *p*-values. **(D)** The boxplot of the 15 functional connections with the lowest *p*-values of PLI (full-frequency band) between the SMCI and PMCI groups. **(E)** The circulargraph of the functional connections with mean PLI (full-frequency band) values exceeding 0.125 within the SMCI and PMCI groups.

As shown in Figure 5, regardless of the functional connectivity features employed, the differences in the functional connectivity in the full-frequency, alpha, theta, and delta bands between SMCI and PMCI patients were more significant than those in the beta and gamma bands. The functional connectivity of full-frequency and alpha bands exhibited better discriminant performance between the two groups, followed by the theta and delta bands.

Also, Figure 5 exhibits the 15 functional connections that yielded the lowest *p*-values for PLI of the full-frequency band between the SMCI and PMCI cohorts. The connectivity strength in the SMCI group was notably higher than in the PMCI group. The SMCI group exhibited a significantly greater number of connections compared to the PMCI group when applying a threshold of 0.125.

### 3.4 Discriminant performance

Table 2 illustrates the discriminant performance of eight classifiers using the previously extracted features between the SMCI and PMCI groups. It can be observed that the KNN exhibited the best classification performance. It had the highest mean and the lowest standard deviation for all evaluation metrics, with an average ACC of 99.96%, AUC of 99.97%, SEN of 99.98%, SPE of 99.95%, PPV of 99.93%, and F1-score of 99.96%. The SVM, LDA, and LogReg fell into the second tier, with the mean for each metric surpassing 95%. The DT, ADA, and RF exhibited slightly lower classification performance, with the mean for each metric remaining above 80%. The NB showed inferior classification performance, although its lowest metric exceeded 75%. The boxplots of discriminant results by different classifiers using 10-fold CV can be found in Appendix B.

**Table 2:** The discriminant results using 10-fold CV with 2 s epochs (mean ± standard deviation, %).

Classifier	ACC	AUC	SEN	SPE	PPV	F1-score
SVM	95.35 ± 2.11	99.12 ± 0.67	94.32 ± 3.81	95.59 ± 3.21	95.23 ± 2.85	94.73 ± 2.62
DT	84.93 ± 3.58	84.68 ± 4.12	83.35 ± 7.17	85.02 ± 6.13	82.84 ± 6.64	82.90 ± 5.64
NB	77.65 ± 5.19	93.38 ± 2.54	85.84 ± 14.59	76.13 ± 16.04	75.25 ± 16.82	77.30 ± 5.68
LDA	96.23 ± 1.87	99.37 ± 0.47	95.38 ± 3.81	96.40 ± 3.04	96.20 ± 2.48	95.73 ± 2.34
ADA	90.58 ± 2.96	96.66 ± 1.48	88.31 ± 5.44	91.50 ± 5.07	90.34 ± 4.22	89.20 ± 3.80
RF	88.87 ± 3.22	98.13 ± 1.11	81.47 ± 13.85	90.97 ± 12.20	94.06 ± 7.02	86.13 ± 5.69
**KNN**	**99.96 ± 0.18**	**99.97 ± 0.16**	**99.98 ± 0.16**	**99.95 ± 0.27**	**99.93 ± 0.40**	**99.96 ± 0.21**
LogReg	95.59 ± 1.84	98.39 ± 1.22	94.65 ± 3.26	95.94 ± 2.91	95.46 ± 2.89	95.01 ± 2.32

Bold values indicates the best discriminant performance.

## 4 Discussion

Based on EEG spectral, nonlinear, and functional connectivity features, we proposed a discriminant framework utilizing machine learning methods to diagnose SMCI and PMCI through computer-aided techniques. We achieved satisfactory classification performance by our data.

The differences in PSD ratio3 and PSD ratio1 between the two groups are pronounced, revealing a distinct “high to low” EEG frequency shift in PMCI patients compared to SMCI patients. This finding provides novel and robust longitudinal evidence for the association between the tendency of change in PSD ratio features and clinical outcomes in aMCI patients, in line with relevant research findings (Luckhaus et al., 2008; Ding et al., 2022; Sadegh-Zadeh et al., 2023). However, the differences in IA between the two groups are not pronounced, suggesting minimal disparities in the distribution of PSD values across the bilateral symmetrical regions of the brain for each frequency band, and requesting further longitudinal evidence. The extracted nonlinear features indicate that the complexity of EEG in the PMCI group is lower than that in the SMCI group. Additionally, the classification performance of SE and PE is superior to that of AE, LZ, and the Hurst exponent. This further underscores that nonlinear features that exhibit outstanding discriminant performance among the AD, MCI, and HC populations may not necessarily apply to distinguishing between the SMCI and PMCI groups (Ruiz-Gomez et al., 2018; Araujo et al., 2022; Ding et al., 2022; Lee et al., 2022). Additionally, we have introduced the innovative nonlinear feature, M-DCPSR, which exhibits significant differences between the two groups and holds promising potential for EEG studies involving aMCI patients or “HC-subjective cognitive decline (SCD)-MCI-AD” spectrum. Significant disparities in functional connectivity were noted between the two groups in both the full frequency and alpha bands, suggesting that the PMCI group exhibits early-stage reductions in intra- and inter-brain region communication during the aMCI phase (Vecchio et al., 2018; Miraglia et al., 2020). Our study showed that the collection of EEG features at the aMCI stage and their follow-up in future studies may crucial for for personalized and precise prevention and treatment strategies.

By comprehensively extracting EEG features, our discriminant framework utilizing machine learning methods has displayed exceptional performance in distinguishing between SMCI and PMCI cases. Notably, all six metrics surpassed 99% in KNN, while all eight classifiers exhibited ACC surpassing 75% and AUC exceeding 80%. These results underscore the value of EEG in automated diagnosis and AD prediction. As KNN excels in handling feature sets with significant dependent, and performs better when the class distributions exhibit distinct clustering characteristics within the feature space (Hu et al., 2022), it outperformed other methods in our research. In contrast, NB relies on the assumption of independence between features (Taheri et al., 2014), which is clearly not met in our research. The observed results may be attributed to the use of PCA for feature dimensionality reduction prior to inputting the data into the machine learning model, aiming to reduce the correlation among the original features. However, while PCA ensures linear independence among the principal components, it does not rule out the possibility of nonlinear relationships.

Given the limited prior application of machine learning methods for longitudinal classification studies involving the aMCI population, we concurrently selected machine learning studies that utilized aMCI as one of their classification labels for comparison with our results (see Table 3). It can be observed that the discriminant framework in this study achieved the highest ACC among all similar studies, indicating significant potential for its application in automated diagnosis and early prediction. Furthermore, using the same discriminant framework, we classified the whole 20-s EEG signals of 107 aMCI patients (results shown in Appendix B). Despite a slight performance decrease, most performance evaluation metrics still exceeded 75%, confirming the stability of our machine learning discriminant framework. The superior classification performance of 2-s epochs compared to 20-s signals in this study may stem from the ability of shorter segments to provide higher temporal and spectral resolution. Differences in frequency domain features between PMCI and SMCI groups likely contributed to this result. We recommend that future studies employing machine learning for EEG analysis report both epoch-based and subject-based classification results whenever possible.

**Table 3:** Comparison between our proposed framework and previous studies (resting-state EEG).

Studies	Subjects	Duration of EEG signal	EEG features	Classifiers	Accuracy (%)	Validation
Vecchio et al. (2018)	74 SMCI, 71 PMCI	2-s	SW property	polynomial regression	61.00	10-fold cross-validation
Li et al. (2021)	28 aMCI, 21 HC	1-s	Graph theory	SVM	86.60	10-fold cross-validation
Li et al. (2022)	26 aMCI, 20 HC	4-s	Spectral entropy	CNN	94.64	10-fold cross-validation
Kim et al. (2022)	139 aMCI, 58 non-aMCI	2-s	Graph theory	LogReg, SVM, RF, NB, GB, NN	89.00	train-test split of 3:7
Youssef et al. (2021)	43 aMCI, 51 HC	4-s	Graph theory	DT	87.20	Leave-one-out cross validation
Höller et al. (2017)	71 aMCI, 39 AD	3-min	Graph theory	SVM	60.00	10-fold cross-validation
Our study	65 SMCI, 42 PMCI	2-s	Spectral, nonlinear, and functional connectivity	SVM, DT, NB, LDA, ADA, KNN, RF, LogReg	**99.96**	10-fold cross-validation

SW, small world; CNN, convolutional neural network; WPLI, weighted phase lag index; GB, gradient boosting; NN, neural network. Bold values indicates the best accuracy.

From the perspective of early prediction, we established a machine learning discriminant framework for SMCI and PMCI using EEG features, achieving remarkable classification performance. However, our study still has several limitations. Firstly, the sample size is relatively small, as all cases were recruited from the First Affiliated Hospital of SYSU. Despite our efforts to expand the epochs to 1,070 by segmenting the EEG data and utilizing 10-fold CVto mitigate the risk of overfitting, the small sample size may still affect the stability and generalizability of the models. With limited data, the models may fail to capture all the important patterns within the data, thereby limiting their applicability and performance in real-world settings. We conducted simulation studies of classifiers under different sample size scenarios and calculated sample size from a statistical perspective (see Appendix C). The results indicate that the sample size in our study is sufficient to infer differences in the metrics. However, we advocate that studies applying machine learning methods in the EEG field should estimate sample sizes beforehand to enhance the credibility of the results. It remains essential to further validate the generalizability of our discriminant framework by increasing the sample size. Therefore, we continue to recruit new cases to enlarge this aMCI cohort and plan to conduct a multi-center study intended for external validation. However, we utilized calibration curves for internal validation of the model, demonstrating the relationship between the predicted probabilities and the observed frequencies (see Appendix B). The results indicate outstanding model calibration, with the curves closely aligning with the ideal diagonal line, suggesting that the predicted probabilities in this study accurately reflect the actual likelihood of events. Secondly, we have overlooked the ranking of feature importance though inter-group comparisons have highlighted statistical significance in extracted features between the two groups. In our future work, we will explore the importance of certain features and the discriminant efficiency under various combinations of feature sets. Additionally, we exclusively employed EEG data obtained at the time of the initial diagnosis, although a longitudinal study on the aMCI cases was conducted. It could be crucial to collect multiple EEG recordings for the aMCI cases during follow-up, as this would aid in dynamically monitoring the trends in EEG features within the aMCI population, thus facilitating the development of an adaptive risk model for the progression from aMCI to AD. However, we proposed a computer-aided diagnostic discriminant framework based on machine learning methods, capable of early predicting AD during the aMCI stage, and achieving satisfactory classification performance.

## 5 Conclusion

Aiming to explore the EEG biomarkers with predictive value for AD in the early stages of aMCI, the proposed discriminant framework provided robust longitudinal evidence for the trajectory of the aMCI cases, aiding in the achievement of early diagnosis and proactive intervention.

## Data Availability

The raw data supporting the conclusions of this article will be made available by the authors, without undue reservation.
