# Supplementary material for: An EEG-based framework for automated discrimination of conversion to Alzheimer’s disease in patients with amnestic mild cognitive impairment: an 18-month longitudinal study
Source: Front Aging Neurosci. 2025 Jan 6;16:1470836. doi: 10.3389/fnagi.2024.1470836 (PMC11743677; doi:10.3389/fnagi.2024.1470836)
Supplement: Supplementary file 1 [file Table_1.DOCX]

Equation for nonlinear and functional connectivity features

**1. Nonlinear feature**

1) Multi-scale permutation entropy (PE):

Given a scalar time series, $\left\{ x\left( i \right):1\leq i\leq N \right\}$. Firstly, reconstruct time series:

$X_{i}=\left\{ x\left( i \right),x\left( i+\tau\right),\ldots,x\left( i+\left( m-1 \right)\tau\right) \right\} i=1, 2, \ldots, N-(m-1)\tau$ (A.1)

where $\tau$ is time delay, and *m* is the embedding dimension.

Then, rearrange $X_{i}$ in an increasing order:

$\{x(i+(j_{1}-1)\tau)\leq x(i+(j_{2}-1)\tau)\leq...\leq x(i+(j_{m}-1)\tau)\}$ (A.2)

There are *m*! permutations for *m* dimensions. Each vector $X_{i}$ can be mapped to one of the *m*! permutations.

Next, the probability of the $j_{th}$ permutation occurring $P_{j}$ can be defined as:

$P_{j}=\frac{n_{j}}{\sum_{j=1}^{m!} n_{j}}$ (A.3)

where $n_{j}$ is the number of times the $j_{th}$ permutation is occurring.

The permutation entropy of the time series $\{x(i): 1\leq i\leq N\}$ is defined by:

$H_{x}\left( m \right)=-\sum_{j=1}^{m!} P_{j}lnP_{j}$ (A.4)

when the time series is random, the $H_{x}\left( m \right)$ approaches its maximum value of ln(*m*!); when the time series is regular, the $H_{x}\left( m \right)$ approaches to zero.

Finally, normalizing $H_{x}\left( m \right)$ by dividing ln(*m*!):

$PE=\frac{H_{x}\left( m \right)}{ln(m!)}$ (A.5)

Multi-scale entropy (MSE) builds on the calculation of entropy by integrating a coarse graining procedure which affords insight into the point-to-point fluctuations over a range of time scales. MSE is calculated as follows:

$y_{j}^{\tau}=\frac{1}{\tau}\sum_{i=\left( j-1 \right)\tau+1}^{j\tau} x_{i} ,1\leq y_{j}\leq\frac{N}{\tau}$ (A.6)

where $\tau$ is the timescale of interest, $y_{j}$ is a data point in the newly constructed time series, $x_{i}$ is a data point in the original time series and *N* is the length of the original time series.

Our study calculated the PE for scales ranging from 1 to 10.

2) Multi-scale approximate entropy (AE):

Approximate entropy is computed in the following way: Firstly, the approximate entropy function generates a delayed reconstruction $Y_{1:N}$ for *N* data points with embedding dimension *m*, and lag $\tau$. Secondly, calculates the number of within range points, at point $i$, given by,

$N_{I}=\sum_{i=1,i\neq k}^{N} l(\left\| Y_{i}-Y_{k} \right\|_{\infty}<r)$ (A.7)

where *l* is the indicator function, and *r* is the radius of similarity. Then, the approximate entropy is calculated as

$AE=\Phi_{m}-\Phi_{m+1}$ (A.8)

where

$\Phi_{m}={(N-m+1)}^{-1}\sum_{i=1}^{N-m+1} log(N_{i})$ (A.9)

Our study calculated the AE for scales ranging from 1 to 10.

3) Multi-scale sample entropy (SE):

Assume we have a time-series data set of length $N=\{x_{1},x_{2},\ldots,x_{N}\}$ with a constant time interval $\tau$*.* We define a template vector of length *m*, such that $X_{M}\left( i \right)=\{x_{i},x_{i+1},x_{i+2},\ldots,x_{i+m-1}\}$ and the distance function $\left\| X_{m}\left( i \right),X_{m}\left( j \right) \right\| (i\neq j)$ is to be the Chebyshev distance. We define the SE to be

$SE=-ln\frac{A}{B}$ (A.10)

where A = number of template vector pairs having $\left\| X_{m+1}\left( i \right),X_{m+1}\left( j \right) \right\|<r$, and B = number of template vector pairs having $\left\| X_{m}\left( i \right),X_{m}\left( j \right) \right\|<r$. We take the value of *m* to be 2 and the value of *r* to be 0.2$\times$Std.

Our study calculated the SE for scales ranging from 1 to 10.

4) Lempel-Ziv complexity (LZ):

LZ is calculated in two steps. Firstly, the value of a given signal is binarized according to its mean value and turning each data point above it to 1 and each point below it to 0. As a second step, the resulting binary sequence is scanned sequentially looking for distinct structures or patterns, building up a dictionary that summarizes the sequences seen so far. Finally, the LZ index is determined by the length of this dictionary. The specific calculation process can be found in Figure 1. Note that regular signals can be characterized by a small number of patterns and hence have low LZ complexity, while irregular signals require long dictionaries and hence have a high LZ complexity.


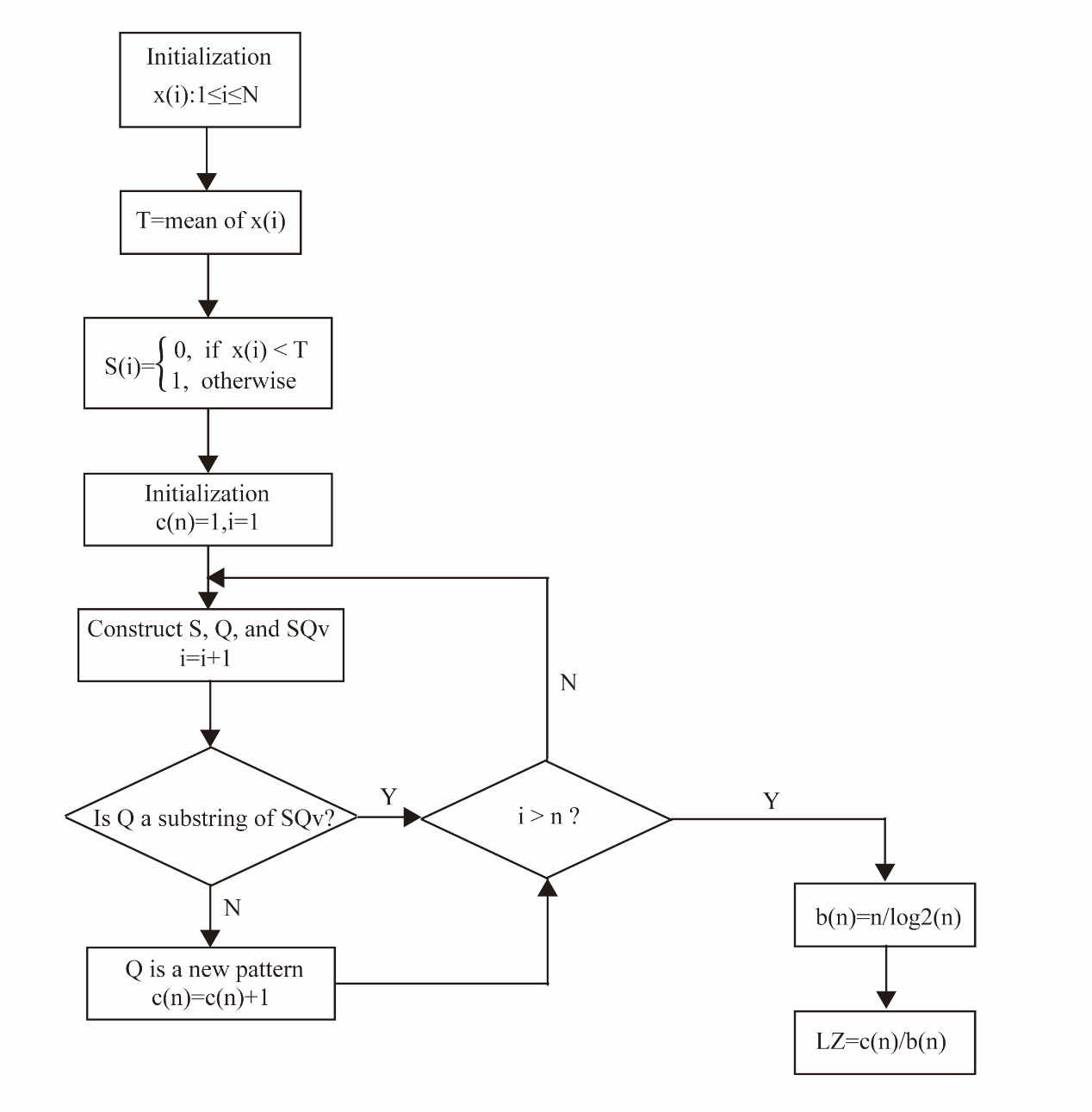


**Supplementary Figure 1** The computation process of LZ

5) Hurst exponent:

Assuming there is a time series of length *N*, denoted as *X*(*t*), where *t* represents the time index from 1 to *N*, Hurst exponent was calculated as following:

5.1) Compute the average of the time series:

$M\left( t \right)=\frac{1}{N}\sum_{i=1}^{N} X(i)$ (A.11)

5.2) Subtract the corresponding scale's mean from each data point:

$Y\left( t,n \right)=X\left( t \right)-M(t-n)$ (A.12)

5.3) Accumulate the deviations over time:

$Z\left( t,n \right)=\sum_{i=1}^{t} Y(i,n)$ (A.13)

5.4) Compute the average of the cumulative deviation:

$R\left( n \right)=\frac{1}{N-n+1}\sum_{t=n}^{N} Z(t,n)$ (A.14)

5.5) Compute the standard deviation of the cumulative:

$S\left( n \right)=\sqrt{\frac{1}{N-n+1}\sum_{t=n}^{N} \left[ Z\left( t,n \right)-R(n) \right]^{2}}$ (A.15)

5.6) Determine the relationship between the standard deviation and the scale, often using a log-log plot, where the slope corresponds to the Hurst exponent:

$H=\frac{log(S(n))}{log(n)}$ (A.16)

6) Median distance from the centroid of phase space reconstruction (M-DCPSR):

Assume we have a time-series data set of length $N=\left\{ x_{1},x_{2},\ldots,x_{N} \right\}$，the calculation is as follows:

6.1) Set the embedding dimension of PSR as *m*=3 and determined the lag of the time series ($\tau$) using the autocorrelation function:

$N=N-x_{mean}=\left\{ x_{1}-x_{mean},x_{2-x_{mean}},\ldots,x_{N}-x_{mean} \right\}$ (A.17)

$SSd=N\cdot N$ (A.18)

$R_{xx}(\tau)=\frac{1}{SSd}\sum_{i=1}^{N-\tau} N(i)\cdot N(i+\tau)$ (A.19)

By detecting zero crossings of the autocorrelation function, the time delay $\tau$ is identified. The values of the autocorrelation function are iterated through in a loop, and when

$R_{xx}\left( j-1 \right)\cdot R_{xx}\left( j \right)\leq0$ (A.20)

, it indicates a zero crossing in the autocorrelation function. Subsequently, the time delay $\tau$ is determined based on the absolute values of the autocorrelation function before and after the crossing point:

$\left\{ \begin{aligned} \tau=j, \left| R_{xx}\left( j-1 \right) \right| \leq\left| R_{xx}\left( j \right) \right| \\ \tau=j-1, \left| R_{xx}\left( j-1 \right) \right| > \left| R_{xx}\left( j \right) \right| \end{aligned} \right.$ (A.21)

6.2) Construct the three-dimensional coordinates of the time series in the phase space based on the τ:

$(x_{n},y_{n},z_{n})=\left\{ \begin{matrix} x_{1} & x_{1+\tau} & x_{1+2\tau} \\ x_{2} & x_{2+\tau} & x_{2+2\tau} \\ \begin{matrix} \vdots\\ x_{N-2\tau} \end{matrix} & \begin{matrix} \vdots\\ x_{N-\tau} \end{matrix} & \begin{matrix} \vdots\\ x_{N} \end{matrix} \end{matrix} \right\}$ (A.22)

each column of matrix represents one dimension of a three-dimensional coordinate.

6.3) Calculate the centroid of the structure formed by all points in the phase space:

$\left( \frac{\sum_{i=1}^{n} x_{i}}{n},\frac{\sum_{i=1}^{n} y_{i}}{n},\frac{\sum_{i=1}^{n} z_{i}}{n} \right)$ (A.23)

6.4) Calculate the Euclidean distance between each point and the centroid:

$d_{n}=\sqrt{\left( x_{n}-\frac{\sum_{i=1}^{n} x_{i}}{n} \right)^{2}+\left( y_{n}-\frac{\sum_{i=1}^{n} y_{i}}{n} \right)^{2}+\left( z_{n}-\frac{\sum_{i=1}^{n} z_{i}}{n} \right)^{2}}$ (A.24)

6.5) Finally, calculate the median of these Euclidean distances, yielding the M-DCPSR for the given time series.

**2. Functional connectivity feature**

1) Correlation coefficient:

The equation for calculating *r* between two signals *X* and *Y* is:

$r=\frac{\sum_{i=1}^{n} (X_{i}-\bar{X})(Y_{i}-\bar{Y})}{\sqrt{\sum_{i=1}^{n} {(X_{i}-\bar{X})}^{2}\sum_{i=1}^{n} {(Y_{i}-\bar{Y})}^{2}}}$ (A.25)

Where *n* is the number of data points, $\bar{X}$ and $\bar{Y}$ are the means of signals *X* and *Y*, respectively.

2) Phase lag index (PLI):

Suppose the band-pass filtered signals at two electrodes are *X*(*t*) and *Y*(*t*), respectively. Through the Hilbert transform, we can obtain their analytic signals $X_{an}(t)$ and $Y_{an}(t)$, as

$X_{an}\left( t \right)=X\left( t \right)+iX_{H}(t)$ (A.26)

$Y_{an}\left( t \right)=Y\left( t \right)+iY_{H}(t)$ (A.27)

where $X_{H}(t)$ and $Y_{H}(t)$ are the Hilbert transform of band-pass filtered signals *X*(*t*) and *Y*(*t*), respectively. Namely,

$X_{H}\left( t \right)=\frac{1}{\pi}P.V.\int_{-\infty}^{+\infty} \frac{X(\tau)}{t-\tau}d\tau$ (A.28)

$Y_{H}\left( t \right)=\frac{1}{\pi}P.V.\int_{-\infty}^{+\infty} \frac{Y(\tau)}{t-\tau}d\tau$ (A.29)

where *P. V.* is the Cauchy principal value.

Using analytical signals, the instantaneous amplitude $A_{x}\left( t \right)$, $A_{y}\left( t \right)$ and instantaneous phase $\phi_{x}\left( t \right)$, $\phi_{y}\left( t \right)$, can be calculated:

$A_{x}\left( t \right)=\sqrt{{X_{an}\left( t \right)}^{2}+{X_{H}\left( t \right)}^{2}}$ (A.30)

$A_{y}\left( t \right)=\sqrt{{Y_{an}\left( t \right)}^{2}+{Y_{H}\left( t \right)}^{2}}$ (A.31)

$\phi_{x}\left( t \right)={tan}^{-1}\frac{X_{H}\left( t \right)}{X_{an}\left( t \right)}$ (A.32)

$\phi_{y}\left( t \right)={tan}^{-1}\frac{Y_{H}\left( t \right)}{Y_{an}\left( t \right)}$ (A.33)

We calculate $\Delta\phi_{xy}\left( t \right)$, which is their phase difference at time *t*:

$\Delta\phi_{xy}\left( t \right)=\left| \phi_{x}\left( t \right)-\phi_{y}\left( t \right) \right|$ (A.34)

In actual analysis, the phase difference needs to be converted to [0, 2π):

$\Delta\phi_{rel}\left( t \right)=\Delta\phi_{xy}\left( t \right)mod2\pi$ (A.35)

Finally, the formula of PLI is:

$PLI=\left| \frac{1}{N}\sum_{N=1}^{N} sign(\Delta\phi_{rel}\left( t \right)) \right|$ (A.36)

3) Magnitude Squared Coherence (MSC):

Suppose *X*(*t*) and *Y*(*t*) represent the EEG signals over electrodes *X* and *Y*, respectively. Firstly, the time domain signals *X*(*t*) and *Y*(*t*) are converted to frequency domain using fast Fourier transform (FFT). Then, for each frequency bin *f*, the individual spectral power density $S_{xx}\left( f \right)$and $S_{yy}(f)$ and their cross spectral power density $S_{xy}(f)$ are estimated. The coherency function $K_{xy}(f)$ is calculated as:

$K_{xy}\left( f \right)=\frac{S_{xy}(f)}{\sqrt{S_{xx}(f)S_{yy}(f)}}$ (A.37)

Lastly, the MSC at frequency bin *f* is computed as:

$MSC=\left| K_{xy}\left( f \right) \right|^{2}$ (A.38)
