# Supplementary material for: An EEG-based framework for automated discrimination of conversion to Alzheimer’s disease in patients with amnestic mild cognitive impairment: an 18-month longitudinal study
Source: Front Aging Neurosci. 2025 Jan 6;16:1470836. doi: 10.3389/fnagi.2024.1470836 (PMC11743677; doi:10.3389/fnagi.2024.1470836)
Supplement: Supplementary file 2 [file Table_2.DOCX]

| **Supplementary Table 1** Number of extracted features | | |
| --- | --- | --- |
| Feature sets | Features | Number of features |
| Spectral | PSD ratio | 5 types $\times$ 16 channels = 80 |
|  | PSD entropy | 5 bands $\times$ 16 channels = 80 |
|  | Interhemispheric asymmetry | 5 bands $\times$ 8 pairs = 40 |
| Complexity | Lempel-Ziv | 16 channels |
|  | Hurst exponent | 16 channels |
|  | M-DCPSR | 16 channels |
|  | Approximate entropy | 10 scales $\times$ 16 channels = 160 |
|  | Sample entropy | 10 scales $\times$ 16 channels = 160 |
|  | Permutation entropy | 10 scales $\times$16 channels = 160 |
| Functional connectivity | Correlation coefficient | 6 bands $\times$ $\binom{16}{2}$pairs = 720 |
|  | PLI | 6 bands $\times\binom{16}{2}$pairs = 720 |
|  | MSC | 6 bands $\times\binom{16}{2}$pairs = 720 |
| PSD: power spectral density; M-DCPSR: Median distance from the centroid of phase space reconstruction; PLI: phase lag index; MSC: magnitude squared coherence; | | |

| **Supplementary Table 2** The discriminant results using 10-fold CV with the whole 20s signal  (mean$\pm$standard deviation, %). | | | | | | |
| --- | --- | --- | --- | --- | --- | --- |
| Classifier | ACC | AUC | SEN | SPE | PPV | F1-score |
| SVM | 78.37$\pm$12.79 | 83.89$\pm$15.63 | 77.68$\pm$19.94 | 76.24$\pm$22.56 | 82.55$\pm$17.91 | 78.47$\pm$14.17 |
| DT | 72.73$\pm$13.31 | 74.94$\pm$15.24 | 75.12$\pm$19.97 | 68.09$\pm$25.49 | 77.18$\pm$18.54 | 73.84$\pm$16.15 |
| NB | 64.75$\pm$16.12 | 75.71$\pm$17.48 | 63.14$\pm$22.97 | 70.66$\pm$26.60 | 74.94$\pm$23.36 | 64.90$\pm$17.38 |
| LDA | 78.50$\pm$12.53 | 83.65$\pm$15.55 | 79.10$\pm$18.57 | 75.15$\pm$23.25 | 80.79$\pm$18.25 | 79.18$\pm$13.93 |
| ADA | 76.10$\pm$12.57 | 82.56$\pm$16.12 | 77.24$\pm$20.24 | 71.21$\pm$26.35 | 79.23$\pm$19.76 | 76.78$\pm$15.36 |
| RF | 75.93$\pm$13.26 | 84.32$\pm$15.43 | 78.38$\pm$22.77 | 67.26$\pm$31.16 | 80.85$\pm$17.38 | 76.83$\pm$15.05 |
| KNN | 75.88$\pm$11.66 | 73.67$\pm$14.07 | 77.83$\pm$19.55 | 69.51$\pm$27.94 | 80.11$\pm$16.39 | 77.00$\pm$13.16 |
| LogReg | 76.94$\pm$13.00 | 81.86$\pm$15.28 | 77.33$\pm$18.77 | 73.64$\pm$24.05 | 79.98$\pm$18.38 | 77.66$\pm$14.21 |

| **Supplementary Table 3** The classifiers and functions used in MATLAB. | |
| --- | --- |
| Classifier | Function |
| SVM | fitcsvm() |
| DT | fitctree() |
| NB | fitcnb() |
| LDA | fitcdiscr() |
| ADA | fitcensemble() |
| RF | TreeBagger() |
| KNN | fitcknn() |
| LogReg | fitglm() |


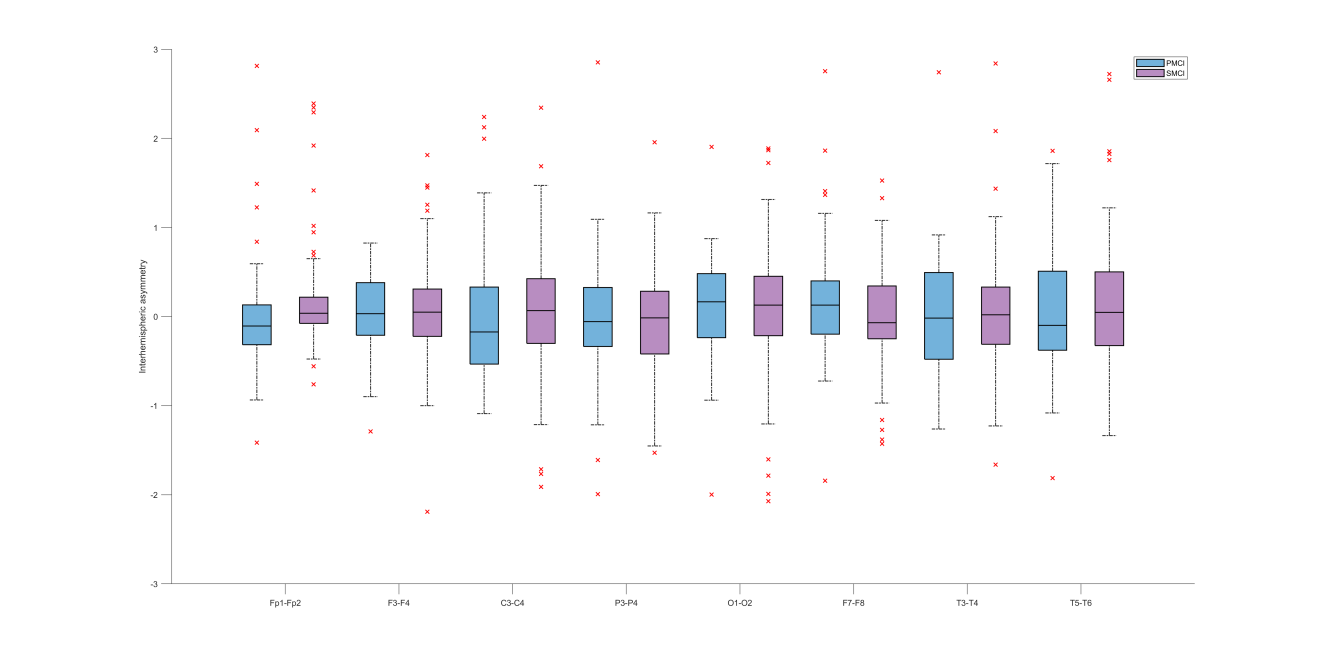


**Supplementary Figure 1** The boxplot of IA in the theta band for the SMCI and PMCI groups. The horizontal axis represents 8 channel pairs, and the vertical axis represents the values of IA.


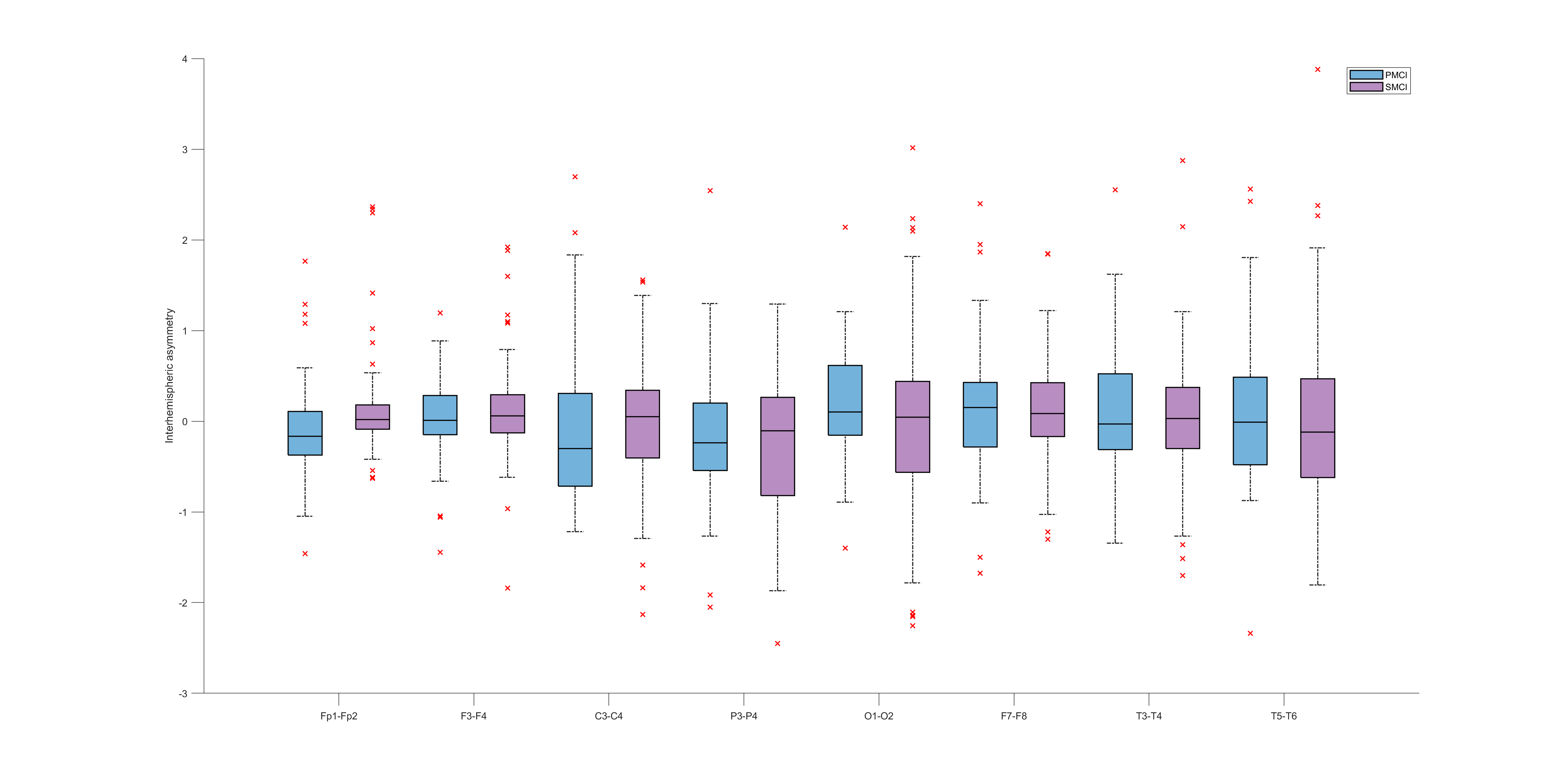


**Supplementary Figure 2** The boxplot of IA in the alpha band for the SMCI and PMCI groups. The horizontal axis represents 8 channel pairs, and the vertical axis represents the values of IA.


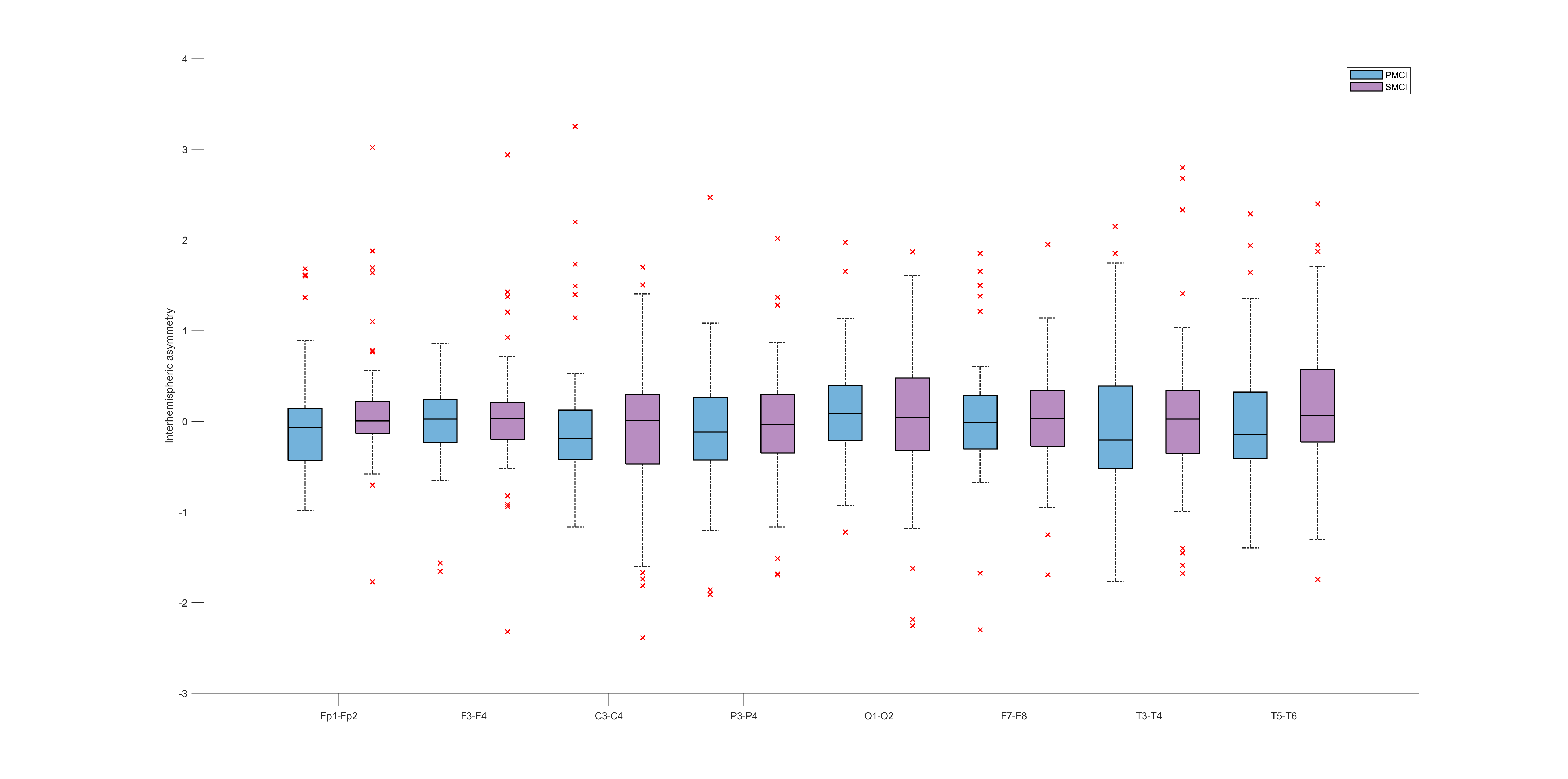


**Supplementary Figure 3** The boxplot of IA in the beta band for the SMCI and PMCI groups. The horizontal axis represents 8 channel pairs, and the vertical axis represents the values of IA.


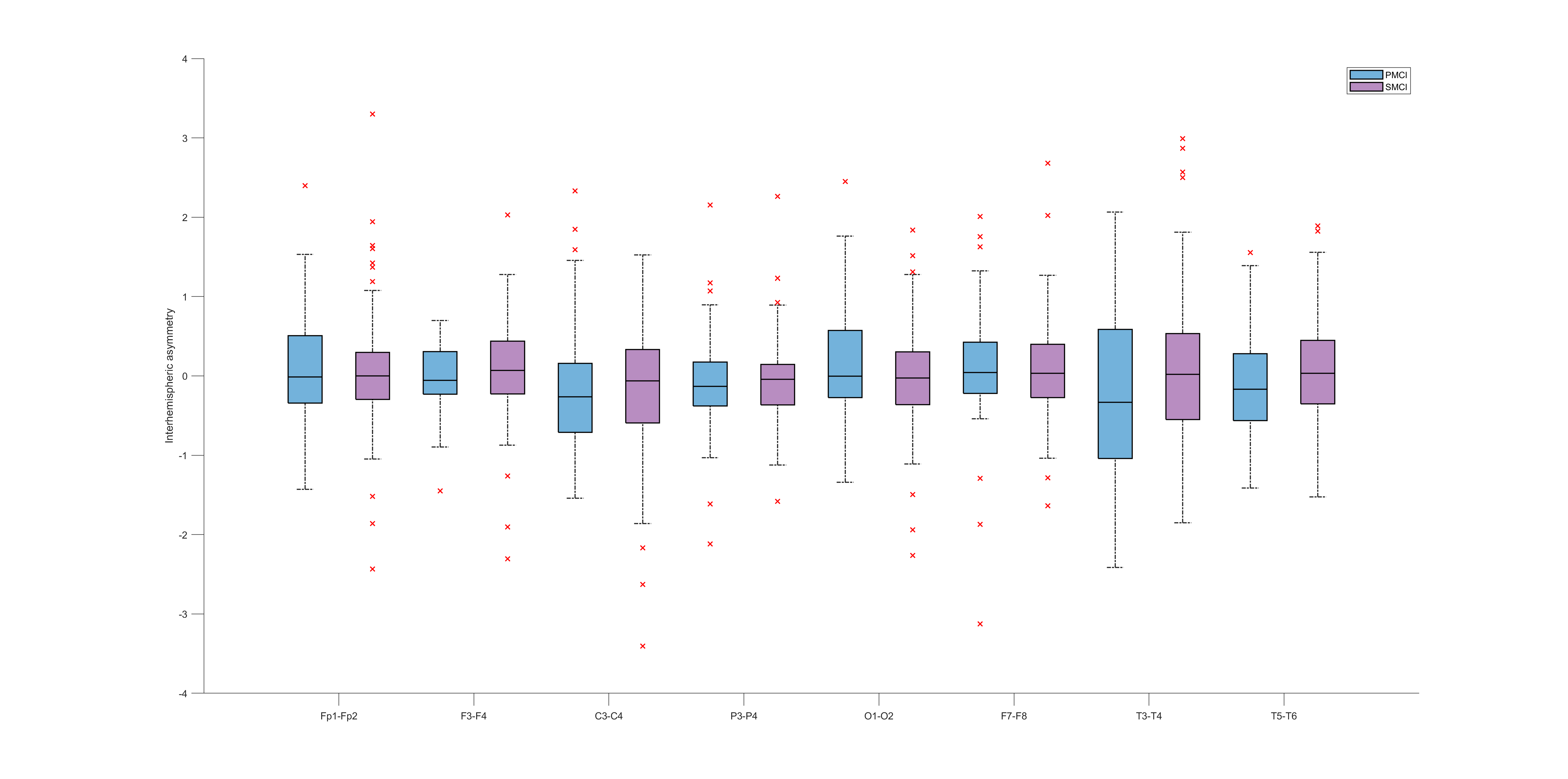


**Supplementary Figure 4** The boxplot of IA in the gamma band for the SMCI and PMCI groups. The horizontal axis represents 8 channel pairs, and the vertical axis represents the values of IA.


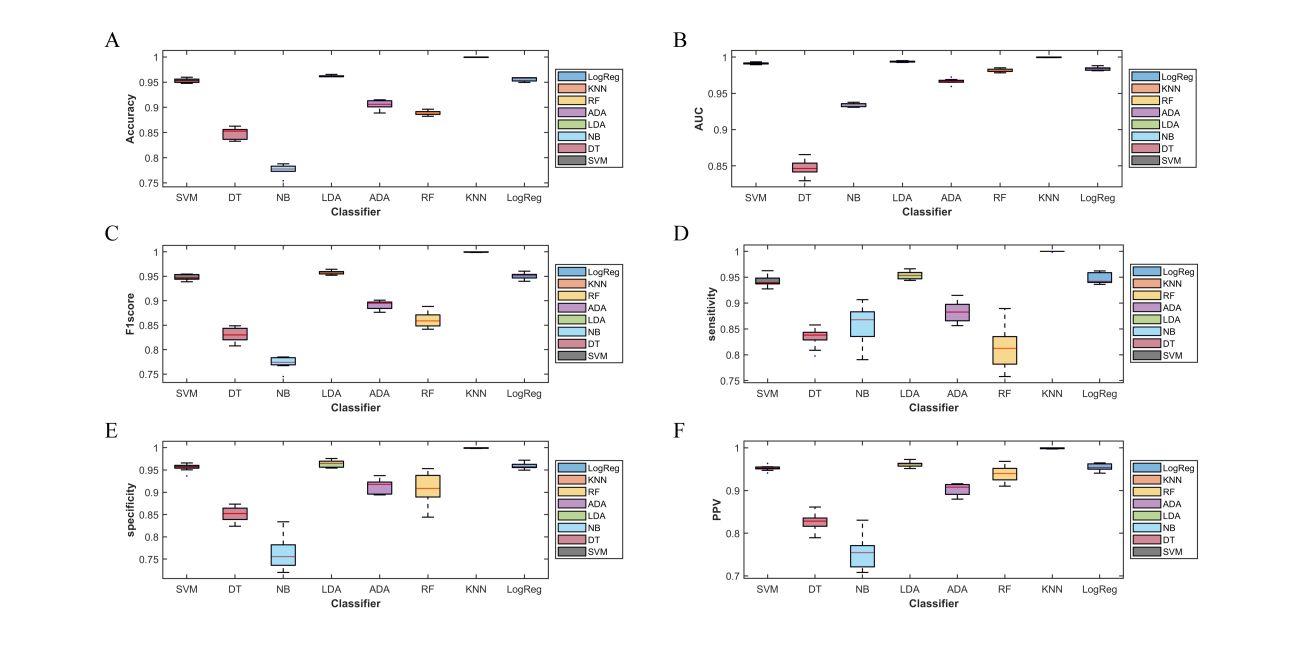


**Supplementary Figure 5** The boxplot of discriminant results by different classifiers using 10-fold CV with 2s epoch in terms of: (A) AC; (B) AUC; (C) F1-score; (D) SE; (E) SP; (F) PPV.


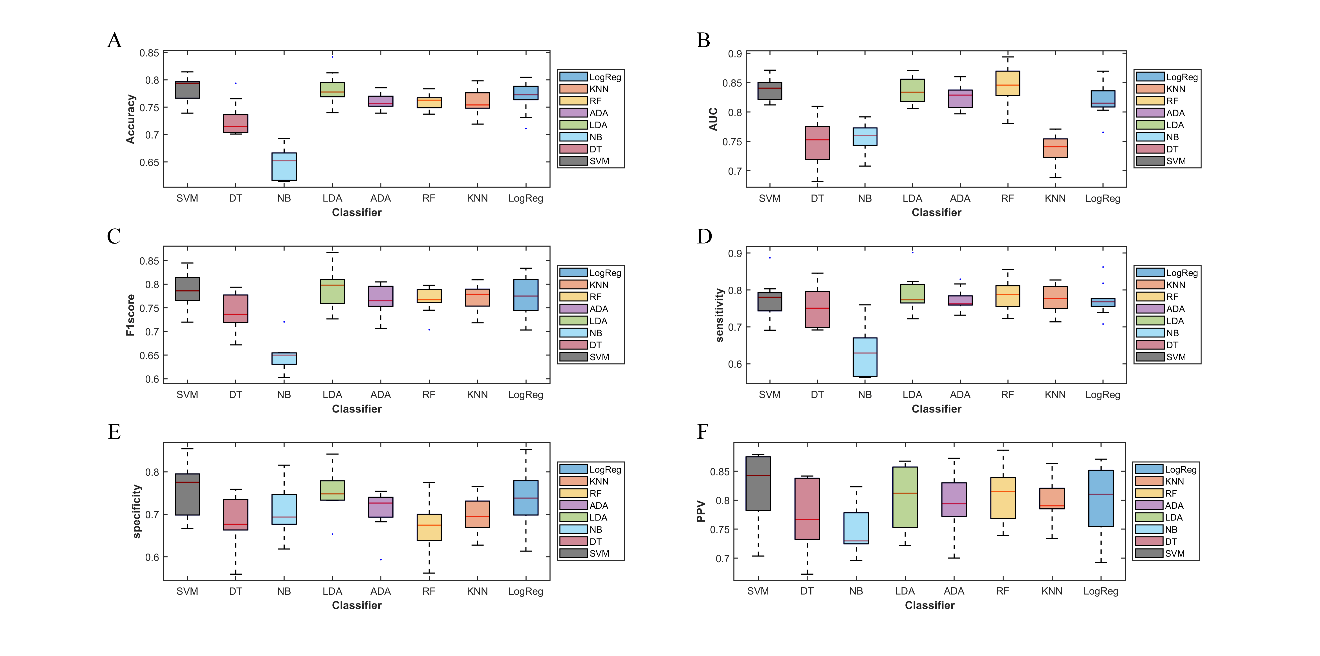


**Supplementary Figure 6** The boxplot of discriminant results by different classifiers using 10-fold CV with the whole 20s signal in terms of: (A) AC; (B) AUC; (C) F1-score; (D) SE; (E) SP; (F) PPV.


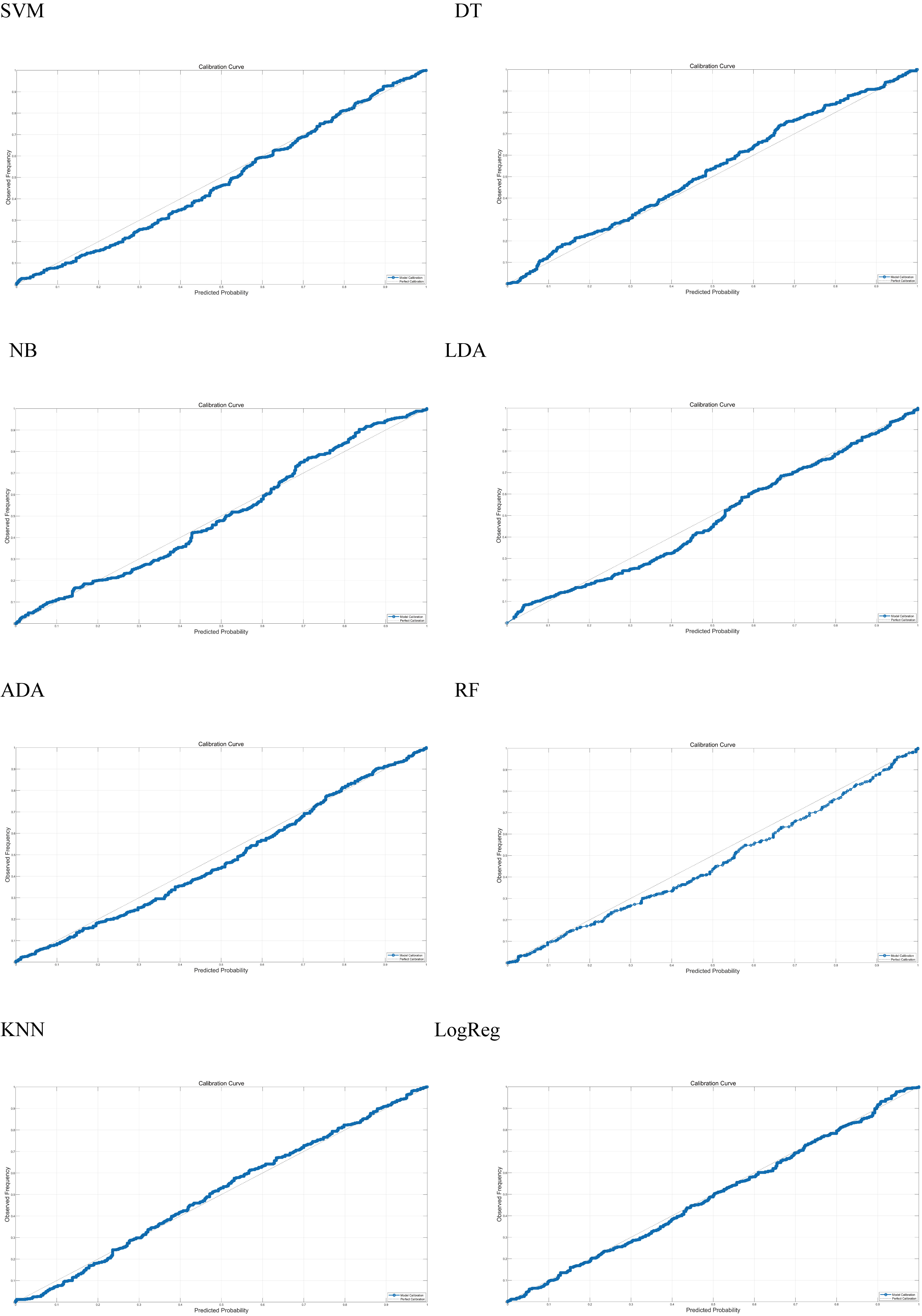
**Supplementary Figure 7** Calibration curves of the classifiers.
