# Supplementary material for: An EEG-based framework for automated discrimination of conversion to Alzheimer’s disease in patients with amnestic mild cognitive impairment: an 18-month longitudinal study
Source: Front Aging Neurosci. 2025 Jan 6;16:1470836. doi: 10.3389/fnagi.2024.1470836 (PMC11743677; doi:10.3389/fnagi.2024.1470836)
Supplement: Supplementary file 3 [file Table_3.DOCX]

A brief introduction to the classifiers

1. Support Vector Machine (SVM): SVM is a supervised learning model that finds the hyperplane that best separates classes in the feature space. It is particularly effective in high-dimensional spaces (Cortes and Vapnik, 1995).

2. Decision Tree (DT): Decision Tree is a non-parametric supervised learning method used for classification and regression. It creates a model that predicts the value of a target variable by learning simple decision rules inferred from the data features. The tree splits data based on feature thresholds to minimize a certain criterion such as Gini impurity or entropy (Quinlan, 1986).

3. Naive Bayes (NB): Naive Bayes is a probabilistic classifier based on Bayes' Theorem, with the assumption that features are conditionally independent given the class label. Despite this simplifying assumption, it is highly efficient and works well for high-dimensional datasets (McCallum and Nigam, 1998).

4. Linear Discriminant Analysis (LDA): LDA is a linear classification technique that finds a linear combination of features which best separates two or more classes. It assumes that each class is normally distributed and has the same covariance matrix, maximizing the ratio of between-class variance to within-class variance (Fisher, 1936).

5. AdaBoost (ADA): AdaBoost is a boosting algorithm that combines multiple weak classifiers, typically decision trees, into a single strong classifier. It assigns weights to each instance, focusing on harder-to-classify examples in subsequent iterations to minimize classification error (Freund and Schapire, 1997).

6. Random Forest (RF): Random Forest is an ensemble learning method based on multiple decision trees. It builds multiple decision trees during training and outputs the class that is the mode of the classes or the average prediction of the individual trees (Breiman, 2001).

7. *k*-nearest neighbor (KNN): KNN is a non-parametric method for classification and regression. For classification, it predicts the class of a data point based on the majority class of its k nearest neighbors in feature space. It is simple and effective but computationally expensive for large datasets (Cover and Hart, 1967).

8. logistic regression (LogReg): Logistic Regression is a linear model used for binary classification. It models the probability that an instance belongs to a particular class using the logistic sigmoid function, optimizing parameters via maximum likelihood estimation (Hosmer and Lemeshow, 2000).

References:

1. Cortes, C., & Vapnik, V. (1995). Support-vector networks. Machine Learning, 20(3), 273–297.

2. Quinlan, J.R. (1986). Induction of Decision Trees. Machine Learning, 1(1), 81–106.

3. McCallum, A., & Nigam, K. (1998). A comparison of event models for Naive Bayes text classification. AAAI Workshop on Learning for Text Categorization, 752, 41–48.

4. Fisher, R.A. (1936). The use of multiple measurements in taxonomic problems. Annals of Eugenics, 7(2), 179–188.

5. Freund, Y., & Schapire, R.E. (1997). A decision-theoretic generalization of on-line learning and an application to boosting. Journal of Computer and System Sciences, 55(1), 119–139.

6. Breiman, L. (2001). Random Forests. Machine Learning, 45(1), 5–32.

7. Cover, T.M., & Hart, P.E. (1967). Nearest neighbor pattern classification. IEEE Transactions on Information Theory, 13(1), 21–27.

8. Hosmer, D.W., & Lemeshow, S. (2000). Applied Logistic Regression. John Wiley & Sons.

Sample size calculation

1. Simulation study

We conducted a simulation study using R (4.3.0), generating 13 different sample size scenarios, each with 63 variables (corresponding to the 63 principal components derived from PCA in our study). We selected the best-performing classifier (KNN) and the least-performing classifier (NB) from our study and employed 10-fold cross-validation to evaluate six performance metrics.

| **Supplementary Table 1** The simulation study of different sample size scenarios for KNN | | | | | | |
| --- | --- | --- | --- | --- | --- | --- |
| Sample size | ACC | AUC | SEN | SPE | PPV | F1-score |
| 50 | 0.76 | 0.75 | 0.57 | 0.92 | 0.87 | 0.68 |
| 100 | 0.77 | 0.77 | 0.82 | 0.72 | 0.75 | 0.78 |
| 200 | 0.80 | 0.79 | 0.86 | 0.71 | 0.81 | 0.83 |
| 300 | 0.79 | 0.77 | 0.91 | 0.63 | 0.76 | 0.82 |
| 400 | 0.80 | 0.80 | 0.87 | 0.73 | 0.77 | 0.82 |
| 500 | 0.79 | 0.73 | 0.86 | 0.70 | 0.78 | 0.82 |
| 600 | 0.74 | 0.74 | 0.86 | 0.61 | 0.71 | 0.77 |
| 700 | 0.75 | 0.74 | 0.90 | 0.58 | 0.71 | 0.79 |
| 800 | 0.76 | 0.74 | 0.95 | 0.53 | 0.71 | 0.81 |
| 900 | 0.79 | 0.79 | 0.82 | 0.76 | 0.78 | 0.80 |
| 1000 | 0.78 | 0.75 | 0.92 | 0.58 | 0.75 | 0.83 |
| 1200 | 0.78 | 0.78 | 0.80 | 0.75 | 0.78 | 0.79 |
| 1500 | 0.78 | 0.78 | 0.86 | 0.69 | 0.77 | 0.81 |

| **Supplementary Table 2** The simulation study of different sample size scenarios for NB | | | | | | |
| --- | --- | --- | --- | --- | --- | --- |
| Sample size | ACC | AUC | SEN | SPE | PPV | F1-score |
| 50 | 0.98 | 0.98 | 1.00 | 0.96 | 0.96 | 0.98 |
| 100 | 0.94 | 0.94 | 0.94 | 0.94 | 0.94 | 0.94 |
| 200 | 0.91 | 0.91 | 0.91 | 0.92 | 0.94 | 0.92 |
| 300 | 0.89 | 0.89 | 0.90 | 0.87 | 0.90 | 0.90 |
| 400 | 0.87 | 0.87 | 0.88 | 0.86 | 0.87 | 0.87 |
| 500 | 0.87 | 0.87 | 0.88 | 0.85 | 0.88 | 0.88 |
| 600 | 0.86 | 0.86 | 0.86 | 0.85 | 0.86 | 0.86 |
| 700 | 0.83 | 0.83 | 0.84 | 0.82 | 0.84 | 0.84 |
| 800 | 0.87 | 0.86 | 0.88 | 0.84 | 0.87 | 0.88 |
| 900 | 0.86 | 0.86 | 0.86 | 0.87 | 0.87 | 0.87 |
| 1000 | 0.86 | 0.85 | 0.90 | 0.80 | .086 | 0.88 |
| 1200 | 0.85 | 0.85 | 0.85 | 0.85 | 0.86 | 0.85 |
| 1500 | 0.86 | 0.86 | 0.89 | 0.82 | 0.86 | 0.87 |

**Codes:**

# Import necessary packages

install.packages("caret")

install.packages("randomForest")

install.packages("e1071")

install.packages("pROC")

install.packages("MLmetrics")

library(caret)

library(randomForest)

library(e1071)

library(pROC)

library(MLmetrics)

# Set random seed for reproducibility

set.seed(123)

# Define sample sizes

sample_sizes <- c(50, 100, 200, 300, 400, 500, 600, 700, 800, 900, 1000, 1200, 1500) # Adjust sample sizes as needed

# Store evaluation results

results_knn <- data.frame(SampleSize = numeric(),

Accuracy = numeric(),

AUC = numeric(),

Sensitivity = numeric(),

Specificity = numeric(),

PPV = numeric(),

F1_Score = numeric())

results_nb <- data.frame(SampleSize = numeric(),

Accuracy = numeric(),

AUC = numeric(),

Sensitivity = numeric(),

Specificity = numeric(),

PPV = numeric(),

F1_Score = numeric())

# Loop through different sample sizes for model training and evaluation

for (n in sample_sizes) {

# Generate simulated data (e.g., for a binary classification problem)

data <- twoClassSim(n)

extra_features <- matrix(rnorm(n * 47), ncol = 47)

colnames(extra_features) <- paste0("Feature", 17:63)

# Define 10-fold cross-validation

train_control <- trainControl(method = "cv", number = 10)

# Train the KNN model

knn_model <- train(Class ~ ., data = data, method = "knn", trControl = train_control)

knn_predictions <- predict(knn_model, data)

# Train the Naive Bayes model

nb_model <- train(Class ~ ., data = data, method = "nb", trControl = train_control)

nb_predictions <- predict(nb_model, data)

# Compute evaluation metrics for KNN

knn_confusion <- confusionMatrix(knn_predictions, data$Class)

knn_roc <- roc(data$Class, as.numeric(knn_predictions))

knn_auc <- auc(knn_roc)

# Compute evaluation metrics for Naive Bayes

nb_confusion <- confusionMatrix(nb_predictions, data$Class)

nb_roc <- roc(data$Class, as.numeric(nb_predictions))

nb_auc <- auc(nb_roc)

# Store results

results_knn <- rbind(results_knn, data.frame(SampleSize = n,

Accuracy = knn_confusion$overall['Accuracy'],

AUC = knn_auc,

Sensitivity = knn_confusion$byClass['Sensitivity'],

Specificity = knn_confusion$byClass['Specificity'],

PPV = knn_confusion$byClass['Pos Pred Value'],

F1_Score = knn_confusion$byClass['F1']))

results_nb <- rbind(results_nb, data.frame(SampleSize = n,

Accuracy = nb_confusion$overall['Accuracy'],

AUC = nb_auc,

Sensitivity = nb_confusion$byClass['Sensitivity'],

Specificity = nb_confusion$byClass['Specificity'],

PPV = nb_confusion$byClass['Pos Pred Value'],

F1_Score = nb_confusion$byClass['F1']))

}

# Display results

print(results)

#-----------------------------------------------------------------------------------------------------

2. Traditional statistical methods

We used the “pwr” package in R to estimate the required sample size. Taking accuracy as the primary outcome, we assumed a baseline accuracy of 0.5 (representing random guessing for a binary classification problem) and an anticipated accuracy of 0.9 for our study. Setting the statistical power at 0.8 and the significance level at 0.05, the required sample size per group was calculated to be 19, or a total of 38 samples.

$n=\frac{{(Z_{\alpha}+Z_{\beta})}^{2}}{h^{2}}$ (C.1)

Where:

*h*: Cohen's *h* (effect size).

$Z_{\alpha}$: The critical value of the standard normal distribution corresponding to the significance level.

$Z_{\beta}$: The critical value of the standard normal distribution corresponding to statistical power.

**Codes:**

# Import the pwr package

install.packages("pwr")

library(pwr)

# Assume the accuracy you want to test (e.g., expected accuracy is 90%)

expected_accuracy <- 0.9

# Assume the baseline accuracy is 50% (accuracy of random guessing)

baseline_accuracy <- 0.5

# Calculate the effect size (Cohen's *h*) to estimate the sample size

# Using the formula *h* = 2 * arcsin(sqrt(p1)) - 2 * arcsin(sqrt(p2))

# p1 is the expected accuracy, p2 is the baseline accuracy

h <- 2 * asin(sqrt(expected_accuracy)) - 2 * asin(sqrt(baseline_accuracy))

# Set the statistical power to 0.8 and the significance level (Alpha) to 0.05

power <- 0.8

alpha <- 0.05

# Calculate the sample size

sample_size <- pwr.2p.test(h = h, power = power, sig.level = alpha)

# Output the sample size

sample_size
